# Supplementary material for: The closed nutrient recycling system in the Paramecium-Chlorella photosymbiosis contributes to survival under oligotrophic conditions
Source: Sci Adv. 2025 Oct 29;11(44):eadz0004. doi: 10.1126/sciadv.adz0004 (PMC12571070; doi:10.1126/sciadv.adz0004)
Supplement: 20251029-1 [file sciadv.adz0004.v1.pdf]

ECOLOGY

Copyright © 2025 The Authors, some rights reserved; exclusive licensee American Association for the Advancement of Science. No claim to original U.S. Government Works. Distributed under a Creative Commons Attribution License 4.0 (CC BY).

The closed nutrient recycling system in the *Paramecium-Chlorella* photosymbiosis contributes to survival under oligotrophic conditions

Kaoru Okada[1,2]*, Takayuki Fujiwara[1,2], Shunsuke Hirooka[2], Yusuke Kobayashi[3], Ryo Onuma[4], Shin-ya Miyagishima[1,2]*

Endosymbiotic relationships between a heterotrophic host and a unicellular algal endosymbiont are observed across many eukaryotic lineages. Although these relationships are prevalent in oligotrophic environments, how they function and provide an advantage under such conditions remains largely unknown. To address these issues, we examined the behavior of the ciliate *Paramecium bursaria* hosting *Chlorella* endosymbionts under nitrogen- and prey-depleted conditions. The *Paramecium* host survived for up to 5 weeks while maintaining the number of *Chlorella* endosymbionts, whereas aposymbiotic *Paramecium* and free-living *Chlorella* either died or bleached, respectively, under the same conditions. In the symbiotic state, the host continuously fed on the endosymbionts without excreting nitrogenous waste into the medium, while the remaining endosymbionts continued to proliferate using heterotrophic metabolites from the host and light energy. Thus, the cyclical farming of endosymbionts by the host maintains a high concentration of nutrients within the closed system, providing a selective advantage in oligotrophic environments.

INTRODUCTION

Endosymbiosis is a process in which a host organism accommodates bacterial or unicellular eukaryotic symbionts within its cells (*1*, *2*). Among several types of endosymbiosis, photosymbiosis—where a heterotrophic eukaryotic host, to utilize their photosynthesis, either harbors cyanobacterial or eukaryotic algal symbionts or temporarily retains chloroplasts from ingested prey—has independently evolved in many unicellular and multicellular eukaryotic lineages (*1*–*3*). For example, several lineages of unicellular eukaryotic hosts, such as ciliates, Centrohelida, amoebozoans, foraminifera, and radiolaria, accommodate unicellular eukaryotic algal endosymbionts (*1*, *3*, *4*). Regarding multicellular organisms, a species of *Hydra* harbors the green alga *Chlorella* as its algal endosymbiont, while giant clams, jellyfish, and cnidarians such as corals harbor dinoflagellate algal endosymbionts (*5*, *6*). In addition, algal chloroplasts, the sites of photosynthesis, themselves originated over a billion years ago when a cyanobacterial endosymbiont became genetically integrated into a eukaryotic host (*7*, *8*). Furthermore, chloroplasts spread to many other lineages through secondary endosymbiosis and the genetic integration of eukaryotic algae into previously heterotrophic eukaryotic hosts (*8*, *9*).

Basically, photosymbiosis functions as a mixotrophic system as a whole, in which the host relies on the photosynthesis of the algal symbiont while also feeding on other microorganisms in the environment (*3*, *10*). The host provides heterotrophic metabolites resulting from the consumption of microbial prey, such as nitrogen sources, to the endosymbionts. In return, the endosymbionts supply the host with photosynthates (*3*, *10*). As a specific example, in the green paramecium (*Paramecium bursaria*), several studies have suggested that the host provides amino acids as nitrogen sources, as well as carbon dioxide derived from its respiration, to the *Chlorella* endosymbiont. In return, the endosymbiont supplies maltose as a source of organic carbon to the host (*11*–*16*). Likewise, in corals, cnidarian hosts supply ammonium and carbon dioxide to the dinoflagellate endosymbionts, while the endosymbionts provide photosynthates such as sugars and glycerol to the hosts (*17*, *18*).

The multiple and independent occurrences of photosymbiosis across diverse lineages raise the question of which environmental pressures have driven its evolution. Regarding this point, photosymbiotic organisms tend to be more prevalent in oligotrophic (nutrient-poor) environments (*19*). For example, photosymbiotic Phaeodaria and Radiolaria (both belonging to the eukaryotic supergroup Rhizaria) that harbor microalgal endosymbionts account for ~50% of the total mesozooplankton biomass (plankton in the size range of 0.2 to 20 mm) in oligotrophic intertropical open oceans (*19*). Similarly, a previous study in an oligotrophic lake reported that ciliates harboring algal endosymbionts made up 60% of the total zooplankton biomass on an annual average (*20*).

On the basis of these observations, uncovering the mechanisms by which photosymbiosis adapts to oligotrophic environments is crucial for understanding its evolution. However, in studies on photosymbiosis to date, organisms have often been cultured under nutrient-rich conditions: *P. bursaria*, as a unicellular model, in media containing high levels of organic components such as plant extracts and abundant microbial prey (*21*, *22*), and hydra and corals, as multicellular models, in prey-rich conditions to maximize their growth rates (*23*, *24*). Thus, the cultivation conditions differ markedly from natural oligotrophic habitats, posing a potential limitation in accurately understanding how photosymbiotic relationships function and confer advantages in nutrient-poor environments.

Under these circumstances, a recent study cultured corals under prey-depleted conditions and showed that the cnidarian host ob-

[1]Department of Genetics, School of Life Science, The Graduate University for Advanced Studies, SOKENDAI, 1111 Yata, Mishima, Shizuoka 411-8540, Japan. [2]Department of Gene Function and Phenomics, National Institute of Genetics, 1111 Yata, Mishima, Shizuoka 411-8540, Japan. [3]College of Science, Graduate School of Science and Engineering, Ibaraki University, Bunkyo, Mito, Ibaraki 310-8512, Japan. [4]Kobe University Research Center for Inland Seas, 2746 Iwaya, Awaji, Hyogo 656-2401, Japan.
*Corresponding author. Email: kokada@nig.ac.jp (K.O.); smiyagis@nig.ac.jp (S.-y.M.)

tains organic compounds containing nitrogen and phosphorus—synthesized by algal endosymbionts as they photosynthetically grow and proliferate within the host—by consuming some of them whole (*25*). The result suggests that, in oligotrophic environments where both inorganic nutrients and prey organisms are scarce, the combination of photosynthetic utilization of inorganic nutrients by the algal endosymbiont and prey consumption by the host gives photosymbiosis a greater advantage than relying on either strategy alone (*25*). As another example, previous studies have shown that *P. bursaria* harboring *Chlorella* endosymbionts is able to survive for an extended period (for a few weeks) under prey-depleted conditions in the light, compared to aposymbiotic *P. bursaria* (*26*, *27*), indicating an advantage of photosymbiosis in prey-limited environments. In addition, a recent study that conducted feeding experiments with various food sources demonstrated that *P. bursaria* harboring *Chlorella* endosymbionts—but not aposymbiotic *P. bursaria*—is able to grow when fed low-quality bacterial food (*28*). Furthermore, another recent study suggested that phagotrophic feeding by the host also benefits the endosymbionts by reducing free-living algal competitors for nutrients essential for photosynthetic growth in the surrounding environment (*29*). However, although photosynthesis by the *Chlorella* endosymbiont is assumed to provide energy for the heterotrophic *Paramecium* host to survive in prey-limited environments (*26*, *27*), previous studies did not examine how the host and endosymbiont behave under such conditions, nor is it clear how energy is transferred from the algal endosymbiont to the host.

To understand how photosymbiotic systems function in oligotrophic environments, we examined the behavior of the *Paramecium* host and *Chlorella* endosymbiont under prey-fed and prey-unfed conditions in this study. Since the growth and photosynthetic activity of algae are known to be rate limited by inorganic nutrients—particularly nitrogen sources (NH_4^+ and NO_3^-), phosphate, and iron—which are scarce in oligotrophic environments (*30*–*33*), we also investigated the behavior of *P. bursaria* under starvation conditions in both inorganic nutrient-replete and nutrient-depleted environments. Here, we show that under starvation, the host feeds on a portion of the algal endosymbionts, while the remaining endosymbionts proliferate through photosynthetic growth, supported by metabolites produced by the host from the digestion of endosymbionts. This enables prolonged host survival and maintains the number of endosymbionts per host cell, even in environments lacking exogenous inorganic nutrients. In addition, we show that the host continues to feed on endosymbionts even while consuming other microbial prey. Thus, the recycling and maintenance of high nutrient concentrations—driven by photosynthesis by the endosymbionts and feeding by the host within the closed host and endosymbiont system—confer an advantage to photosymbiosis in nutrient-limited environments.

RESULTS

Paramecium harboring *Chlorella* endosymbionts survive starvation under light conditions regardless of external nitrogen availability

Several species of freshwater ciliates host green algae of the genus *Chlorella* as endosymbionts (*34*). Among them, *P. bursaria* harbors 300 to 500 *Chlorella variabilis* cells per individual under natural conditions, with each algal cell enclosed by the perialgal vacuolar membrane of the host cell (Fig. 1A) (*35*). Within the *Paramecium* host, *Chlorella* endosymbionts proliferate alongside host growth and are vertically transmitted to daughter cells during host cell division (*36*, *37*). In laboratory cultures with specific treatments, *Paramecium* hosts without *Chlorella* endosymbionts can be generated (*38*), and they proliferate by feeding on microbial prey. Likewise, *Chlorella* cells isolated from the host can also grow independently through photosynthesis (Fig. 2) (*39*).

To examine the behavior of the *Paramecium* host and *Chlorella* endosymbiont under fed and starved conditions, we cultivated *P. bursaria* [NIES-2891, in which a clone of *C. variabilis* (clone A1), isolated from the original strain, was reinfected into an aposymbiotic *P. bursaria* host] in an inorganic liquid medium [modified AF-6 (mAF-6), containing inorganic nitrogen, phosphorus, and iron sources necessary for the photosynthetic growth of algae], supplemented with the green alga *Rusalka fusiformis* as prey, under light conditions. Afterward, the prey was removed from the culture. *P. bursaria* cells were maintained without prey for 2 days to allow complete digestion of the ingested prey, and after replacing the medium with fresh medium (defined as day 0; fig. S1), we examined changes in the number of *Paramecium* cells and the number of *Chlorella* endosymbionts per host cell in the starved cultures under light and dark conditions (Fig. 1 and fig. S2). In addition, to gain insight into whether the behavior of the host and symbiont is related to the oligotrophic conditions in which photosymbiotic organisms prevail (*19*, *20*), starved cultivation was conducted in two types of media: one containing inorganic nitrogen sources (NH_4^+ and NO_3^-) (mAF-6; +N) and one without them (mAF-6ΔN; −N) (Fig. 1).

Consistent with the previous studies (*26*, *27*), the number of *Paramecium* host cells remained almost constant under light conditions, and no dead cells were observed for at least 3 weeks under starvation in the nitrogen-replete inorganic medium (Fig. 1C). Regarding the *Chlorella* endosymbionts, their number per host cell also remained nearly constant during starvation in the light (Fig. 1C). In addition, they retained their green color, and consistent with this observation, the chlorophyll *a* and *b* content per *Chlorella* endosymbiont cell did not decrease but rather increased (approximately twofold) during the starvation culture in the nitrogen-replete medium (fig. S3). However, in the dark, in the nitrogen-replete inorganic medium, the number of *Chlorella* endosymbionts per *Paramecium* host cell continued to decrease during starvation culture, and *Chlorella* cells being digested were observed in the host cells (Fig. 1B). Under this condition, the number of *Paramecium* host cells began to decrease after day 10, and all had died by day 21 (Fig. 1C).

When aposymbiotic *Paramecium* cells (with *Chlorella* endosymbionts removed from *P. bursaria* NIES-2891) were starved in the light or dark in the nitrogen-replete inorganic medium, they began dying immediately after the initiation of starvation, and all had died by day 17 (Fig. 1C and fig. S2). This progression of death under both light and dark conditions was faster than that of the *Paramecium* cells harboring the *Chlorella* endosymbionts starved in the dark (Fig. 1C). The difference in the longevity of the *Paramecium* cells, along with the observation of *Chlorella* endosymbionts being digested within the starved *Paramecium* cells in the dark, suggests that the *Paramecium* host harboring *Chlorella* endosymbionts can survive starvation in the dark longer than in the aposymbiotic state by feeding on *Chlorella* endosymbionts.

When the *Paramecium* cells with or without *Chlorella* endosymbionts were starved in the nitrogen-depleted inorganic medium in the light or dark, the results were similar to those in the nitrogen-containing medium (Fig. 1C). The only difference was that the

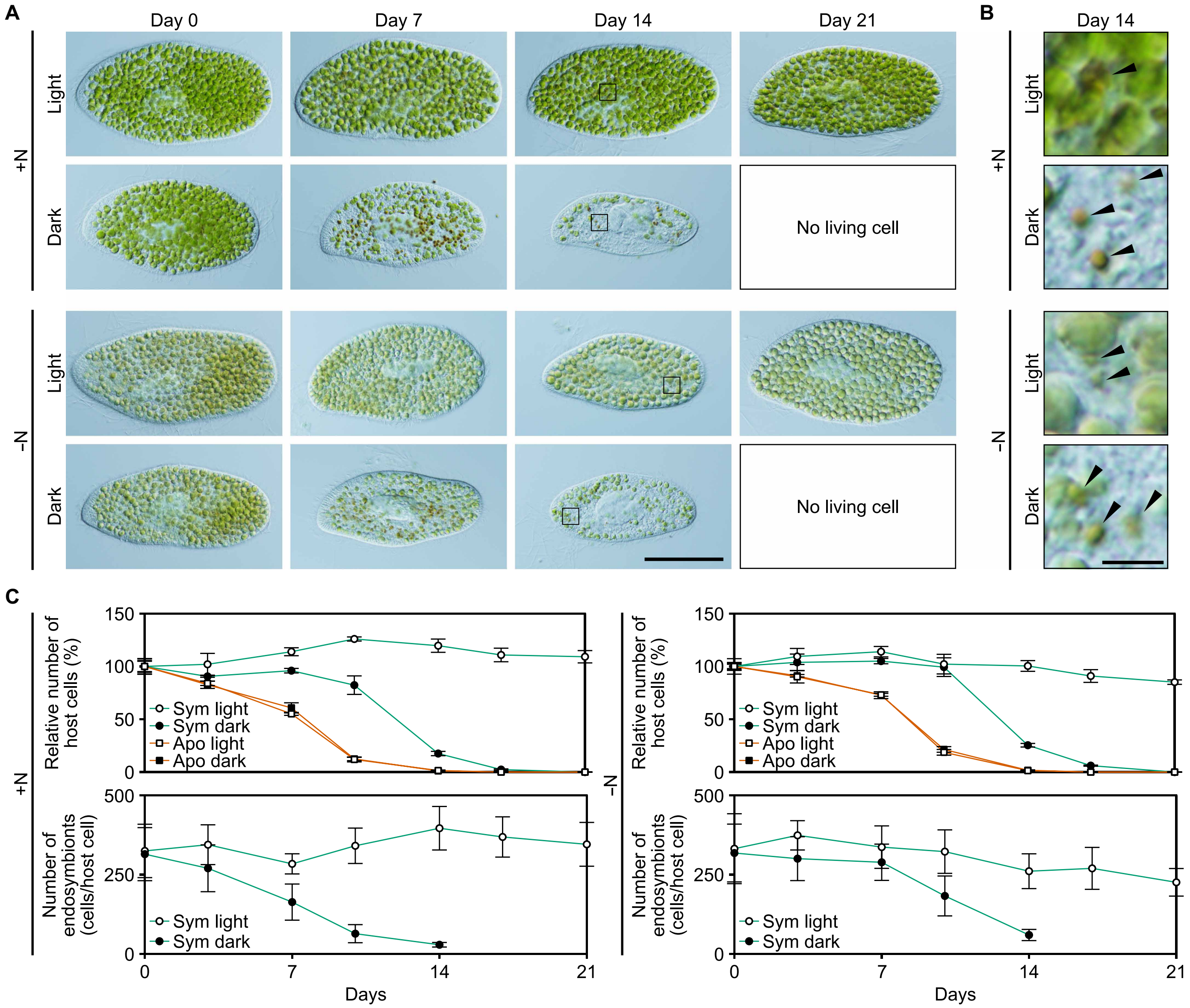

Fig. 1. Changes in morphology and number of *P. bursaria* hosts and *C. variabilis* endosymbionts during starvation in nitrogen-replete or nitrogen-depleted inorganic media. (**A**) Micrographs showing changes in the morphology of *P. bursaria* host cells during starvation. Representative images of *P. bursaria* cells harboring *C. variabilis* endosymbionts under both light and dark conditions in nitrogen-replete (mAF-6; +N) and nitrogen-depleted (mAF-6ΔN; −N) inorganic media are shown. Scale bar, 50 μm. Micrographs of aposymbiotic *P. bursaria* cells are shown in fig. S2. Sym, endosymbiotic; Apo, aposymbiotic. (**B**) Magnified images of *C. variabilis* endosymbionts inside *P. bursaria* host cells, corresponding to the squared regions in (A). Arrowheads indicate endosymbionts being digested. Scale bar, 5 μm. Changes in the ratio of endosymbionts being digested under each condition are shown in fig. S6. (**C**) Changes in the number of *P. bursaria* host cells (expressed as a percentage relative to day 0; 100% corresponds to approximately 200 cells/ml in each culture) and the number of *C. variabilis* endosymbionts per *P. bursaria* host cell during starvation. Means ± SDs from four independent cultures are shown for *P. bursaria*. Means ± SD from five *P. bursaria* cells harboring endosymbionts are shown for *C. variabilis*.

chlorophyll content per *Chlorella* endosymbiont cell increased more in the nitrogen-replete medium (approximately twofold) than in the nitrogen-depleted medium (approximately 1.8-fold) after 3 weeks of starvation in the light (fig. S3). Thus, under starvation, the *Paramecium* host can survive longer than aposymbiotic *Paramecium* while maintaining the number of *Chlorella* endosymbionts, relying on their photosynthesis regardless of external nitrogen availability.

The *Chlorella* endosymbiont bleaches in monoculture in a nitrogen-depleted medium

The above results show that when light is available, the *Chlorella* endosymbionts maintain their numbers and color in the starved *Paramecium* host, even in the absence of nitrogen sources in the medium. However, under nitrogen-depleted conditions, several lineages of algae undergo bleaching as they reallocate nitrogen to molecules other

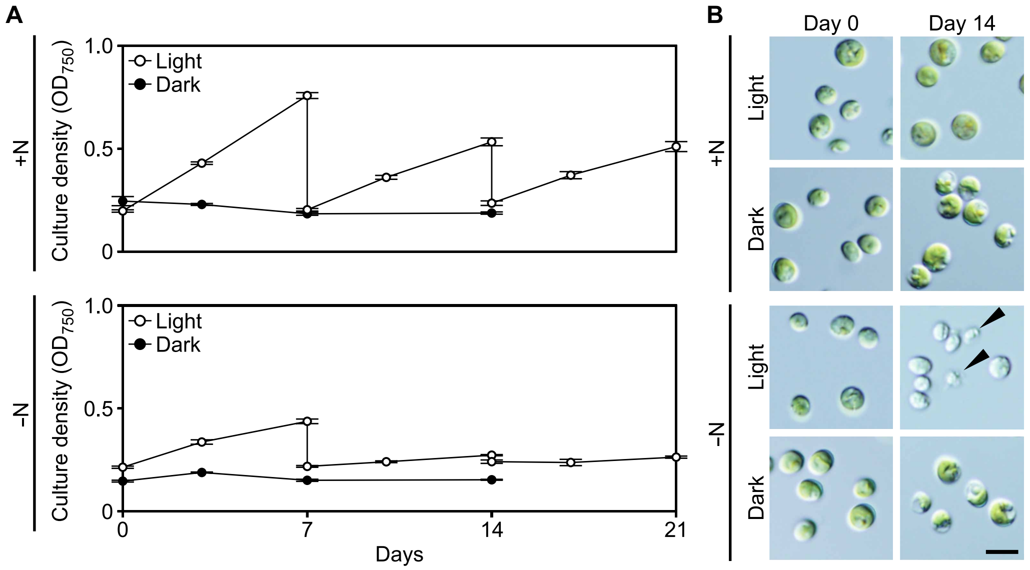

Fig. 2. Changes in the cell density and morphology of monocultured *C. variabilis* in nitrogen-replete or nitrogen-depleted media under light or dark conditions. (**A**) Changes in the cell density (OD_{750}) of cultures in nitrogen-replete (+N) and nitrogen-depleted (−N) inorganic media under light or dark conditions. For cultures in the light, the cells were diluted into fresh medium every 7 days to prevent nutrient depletion and entry into the stationary phase. Means ± SD from four independent cultures are shown. (**B**) Micrographs showing changes in cell morphology and color. The arrowheads indicate broken cells. Scale bar, 5 μm. Changes in cellular chlorophyll concentration are shown in fig. S3.

than photosynthetic pigments and proteins, resulting in the loss of photosynthetic activity (*40*–*42*).

To examine the effect of nitrogen depletion on the *Chlorella* endosymbiont under free-living conditions, we monocultured *C. variabilis* (clone A1) in the light using nitrogen-replete (mAF-6) and nitrogen-depleted (mAF-6ΔN) media (Fig. 2). To prevent the cells from entering the stationary phase due to the consumption of inorganic nutrients in the medium, the cultures under light conditions were diluted into fresh medium on days 7 and 14 (Fig. 2A). In the nitrogen-replete medium, *Chlorella* cells continued to proliferate in the light, maintaining their morphology and cellular chlorophyll *a* and *b* levels (Fig. 2 and fig. S3). In contrast, when *Chlorella* cells were transferred from the nitrogen-replete to the nitrogen-depleted medium, they initially proliferated slowly, approximately doubling in number over 7 days in the light, but after that, they almost ceased proliferation (Fig. 2A). During cultivation in the nitrogen-depleted medium in the light, the cellular chlorophyll level continued to decrease, and by day 14, nearly bleached and broken cells were observed (Fig. 2B and fig. S3). These results are, in principle, the same as those observed in several lineages of free-living algae (*43*, *44*). Combined with the above observations from the *Chlorella* endosymbiont in the *Paramecium* host, these results indicate that the photosymbiotic relationship extends the longevity of both the *Paramecium* host and *Chlorella* endosymbionts, as well as their photosynthetic activity, compared to when they function independently, in conditions where external nitrogen sources are unavailable.

Continuous proliferation of the *Chlorella* endosymbionts in the starved *Paramecium* host suggested by comparative transcriptome analyses

The above results show that in the nitrogen-depleted medium, *Chlorella* cells can survive and maintain their photosynthetic machinery within the starved *Paramecium* host, but not by themselves in monoculture. On the basis of these observations, one possibility arises that the *Chlorella* endosymbiont is supplied with nitrogen sources by the host cell even in the absence of prey and external inorganic nitrogen sources. Regarding this point, previous studies have suggested that when the *Paramecium* host feeds on prey, it supplies *Chlorella* endosymbionts with amino acids rather than inorganic compounds such as NH_4^+ or NO_3^- as nitrogen sources (*12*, *16*).

To gain insights into how the *Chlorella* biomass and photosynthetic machinery are maintained during starvation of the *Paramecium* host, we compared the transcriptomes [RNA sequencing (RNA-seq data)] of the *Paramecium* host (dataset S1) and the *Chlorella* endosymbiont (dataset S2) between fed and starved (day 3) conditions under several cultivation conditions (i.e., with or without external inorganic nitrogen sources or illumination and, for the *Paramecium* host, with or without *Chlorella* endosymbionts; except for fed *P. bursaria* with or without *Chlorella* endosymbionts under dark conditions, which were not analyzed).

In a two-dimensional map of the *Paramecium* host transcriptomes generated by t-distributed stochastic neighbor embedding (t-SNE), a distinct difference was observed between the aposymbiotic (right) and symbiotic (left) groups (Fig. 3A). The aposymbiotic group was further separated into starved (bottom right) and fed (upper right) states (Fig. 3A). In contrast, different endosymbiotic conditions (in fed versus starved hosts; in nitrogen-replete versus nitrogen-depleted media; light versus dark) did not show such distinct differences in positioning (Fig. 3A). We will later return to the interpretation of this distinction based on the results described later.

Regarding the *Chlorella* endosymbiont, the most distinct separation was between light (upper) and dark (bottom right) conditions (Fig. 3B). Through further classification, three groups emerged: (i) in the dark (bottom right), where energy for assimilating nitrogen sources is unavailable to the *Chlorella* endosymbiont; (ii) in the light in the nitrogen-replete medium (top center), the *Chlorella* endosymbiont can potentially assimilate both environmental inorganic

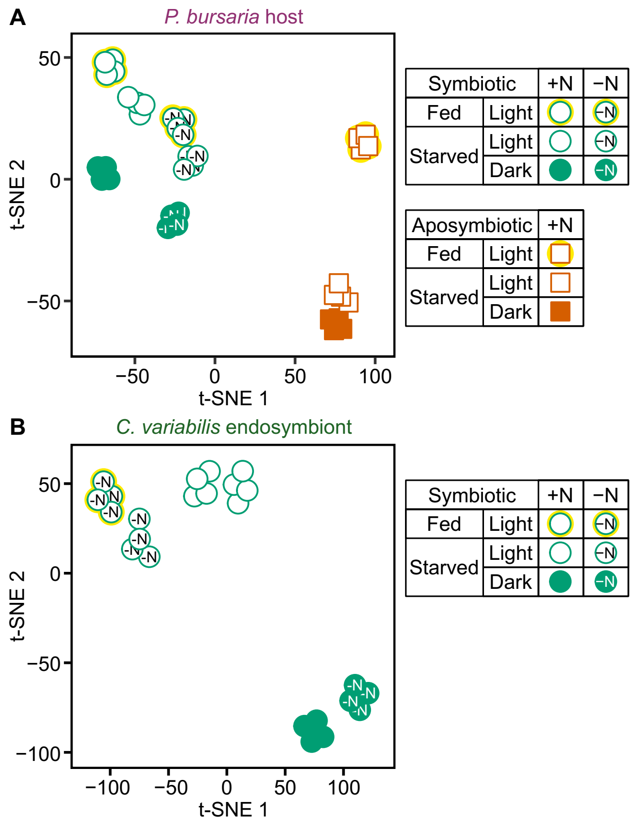

Fig. 3. t-SNE two-dimensional comparison of the transcriptomes of *P. bursaria* host and *C. variabilis* endosymbiont under fed and starved conditions. (**A** and **B**) RNA-seq results from four independent cultures for each condition are shown for both the host and endosymbionts under fed and starved conditions in nitrogen-replete (mAF-6; +N) and nitrogen-depleted (mAF-6ΔN; −N) media under light or dark conditions. For the starved condition, cells were analyzed 3 days after the initiation of the starvation culture. The IDs, read counts, and TPM values of the genes from the RNA-seq analysis are listed in datasets S1 and S2.

nitrogen sources and host-derived organic nitrogen sources (amino acids); (iii) in the light in the nitrogen-depleted medium (upper left), the *Chlorella* endosymbiont can potentially utilize only host-derived organic nitrogen sources (amino acids) (Fig. 3B).

Related to these results, the impact of the presence or absence of inorganic nitrogen sources in the medium on the *Chlorella* endosymbiont was evident. As observed in the responses of several algal species to nitrogen limitation, in the *Chlorella* endosymbiont accommodated in the *Paramecium* host, genes encoding ammonium transporters and enzymes involved in nitrogen assimilation were up-regulated in nitrogen-depleted compared to nitrogen-replete media, regardless of the availability of prey for the host (Fig. 4A and dataset S3).

However, in both the t-SNE maps of *Paramecium* host and *Chlorella* endosymbiont, there was no clear separation between the fed and starved conditions of the *Paramecium* host (Fig. 3A). Specifically, for the *Chlorella* endosymbiont, the transcriptomes in the fed and starved conditions were positioned in nearly the same location, although their positions differed between the nitrogen-replete and nitrogen-depleted media (Fig. 3B). In addition, the differences in

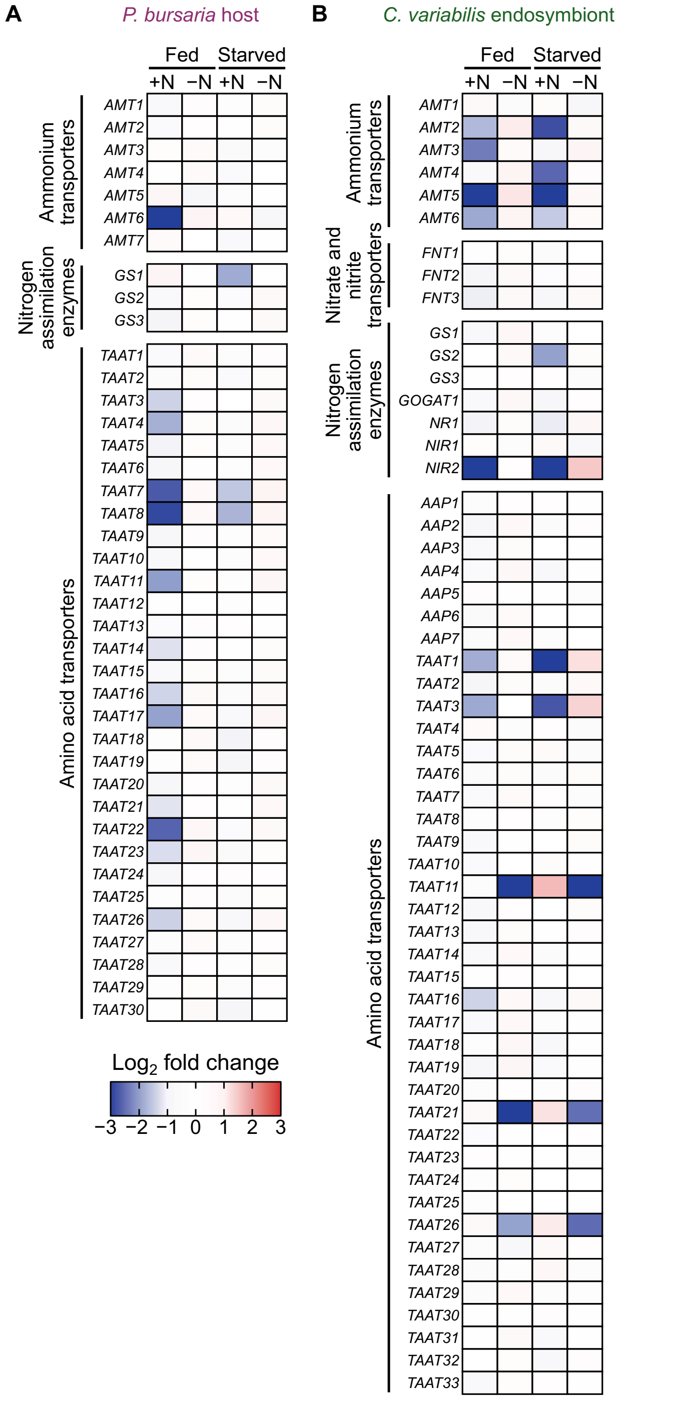

Fig. 4. Effects of inorganic nitrogen or prey availability on expression of nitrogen assimilation and amino acid transporter genes in *P. bursaria* host and *C. variabilis* endosymbiont in the light. (**A** and **B**) RNA-seq results (from four independent cultures per condition) were compared for *P. bursaria* cells harboring *C. variabilis* endosymbionts under light conditions across four conditions: nitrogen-depleted (mAF-6ΔN; −N) or nitrogen-replete (mAF-6; +N) media, with (fed) or without prey (starved). Log_2 fold differences in mRNA levels (TPM) for each gene under the respective conditions, relative to the mean across all four conditions (defined as log_2 fold difference = 0), are shown using color scales for *P. bursaria* host genes (A) and *C. variabilis* endosymbiont genes (B). For the starved condition, cells were analyzed 3 days after the initiation of the starvation culture. The IDs, read counts, and TPM values of the genes from the RNA-seq analysis are listed in datasets S1 to S3.

mRNA levels of host and endosymbiont genes encoding enzymes involved in nitrogen assimilation and amino acid transporters between the fed and starved conditions were slight compared to the differences observed between the nitrogen-replete and nitrogen-depleted media (Fig. 4B and dataset S3). Regarding this point as well, we will later return to the interpretation based on the results described later.

To gain insights into whether, and if so, how nitrogen is supplied to *Chlorella* endosymbiont by the *Paramecium* host under starvation in a nitrogen-depleted medium in the light, we classified functions that were up- or down-regulated upon host starvation according to the Kyoto Encyclopedia of Genes and Genomes (KEGG) database (figs. S4 and S5 and datasets S1, S2, and S4 to S7). As a result, most metabolic pathways were down-regulated in both the *Paramecium* host (fig. S4) and the *Chlorella* endosymbiont (fig. S5) under starvation. We further examined KEGG subcategories (datasets S4 and S5) and annotations of genes notably up-regulated under starvation [$\log_2$ fold change > 1; false discovery rate (FDR) < 0.05] in both the *Paramecium* host (667 genes in nutrient-replete and 274 in nitrogen-depleted media) and the *Chlorella* endosymbiont (1879 genes in nutrient-replete and 1661 in nitrogen-depleted media) (datasets S6 and S7). However, no notable enrichment of pathways was found that would explain how the endosymbiont avoids bleaching when the host is starved and no nitrogen is available from prey or the medium.

Then, we focused on the following possible inconsistencies between the observed changes in host and endosymbiont numbers and the transcriptome data from the fed and starved cultures. In the fed culture under light conditions, both the *Paramecium* host and the *Chlorella* endosymbionts proliferated, while in the starved culture under light conditions, the numbers of both the *Paramecium* host and the *Chlorella* endosymbionts remained unchanged for 21 days (Fig. 1). Consistent with these results, in the KEGG classification of the *Paramecium* host under starved conditions, down-regulated genes were prominent in categories related to cell proliferation ("nucleotide metabolism," "replication and repair," and "cell growth and death") (fig. S4). In contrast, these categories on the *Chlorella* endosymbiont side under starved conditions contained both up- and down-regulated genes, although the number of down-regulated genes was somewhat higher (fig. S5). To explore the cause of this difference, we examined the expression of genes involved in cell cycle progression in the *Paramecium* host and the *Chlorella* endosymbiont, which are expressed only when the cells undergo division.

According to the RNA-seq data (day 3 of the cultures), genes known to be expressed only during the S or M phase in ciliates (*MCM2*, *CYC1*, and *CDK1*) (*45*) in the *Paramecium* host were, as expected, substantially down-regulated in the starved culture in the light, where *Paramecium* did not proliferate but survived, and in the dark, where it gradually died, compared to the fed culture in the light, where it proliferated (Fig. 5A and datasets S1 and S3). In contrast, on the basis of the RNA-seq data, the levels of S phase–specific genes [*PCNA*, *CYCB*, and *FTSZ* (*46*, *47*)] in the *Chlorella* endosymbiont within the starved *Paramecium* host in the light, where the *Chlorella* biomass (i.e., the number of host cells and the number of *Chlorella* endosymbionts per host cell) remained constant, were similar to those in the fed host in the light, where the *Chlorella* biomass increased in accordance with the proliferation of the host (Fig. 5A and datasets S2 and S3). Conversely, the mRNA levels of these *Chlorella* genes substantially decreased in the starved host in the dark, where the number of *Chlorella* cells decreased because of digestion by the host (Fig. 5A and datasets S2 and S3). In addition, quantitative reverse transcription polymerase chain reaction (qRT-PCR) showed that these three cell cycle genes were expressed in proliferating monocultured *Chlorella* in the light but not in nonproliferating cells in the dark (Fig. 5). In the starved *Paramecium* host, these genes were expressed on days 7, 14, and 21 in the *Chlorella* endosymbiont in the light but not in the dark (Fig. 5B). The above-mentioned patterns of cell cycle gene expression in the host and endosymbionts were consistent between the cultures in the nitrogen-replete and nitrogen-depleted media (Fig. 5).

These results raise the possibility that the *Chlorella* endosymbionts continue to proliferate in the starved host in both the nitrogen-replete and nitrogen-depleted media in the light. However, since the total mass of *Chlorella* in the culture (i.e., the number of host cells and the number of *Chlorella* endosymbionts per host cell) remained constant for 21 days in the starved host in the light, it also raises the possibility that the increased number of *Chlorella* endosymbionts is continuously digested by the host as an energy source. Thus, we examined this possibility in the following analyses.

The starved *Paramecium* host feeds on *Chlorella* endosymbionts proliferating in the light

To confirm whether the *Chlorella* endosymbionts continue to proliferate in the starved *Paramecium* host in both nitrogen-replete and nitrogen-depleted media under light conditions, the starved *Paramecium* host harboring the *Chlorella* endosymbionts was subjected to a diurnal 12-hour light/12-hour dark (LD) cycle. As in many other algae, cell cycle progression in *Chlorella* is known to be synchronized with the LD cycle: The cells grow during the daytime and divide at night. Depending on the growth rate, they divide once or twice successively, forming two or four daughter cells within the mother cell wall, which then hatch out (Fig. 6A) (*37*). Thus, if, in the starved *Paramecium* host under the LD cycle, two-cell and four-cell stages of the *Chlorella* endosymbionts periodically accumulate and decrease, then this would indicate that the expression of S or M phase–specific genes observed above (Fig. 5) is not due to abnormalities in cell cycle progression, such as arrest during the S or M phase, but rather that the cells continue to proliferate.

In the starved *Paramecium* under the LD cycle in both nitrogen-replete and nitrogen-depleted media, two-cell and four-cell stages of *Chlorella* endosymbionts accumulated and then disappeared on days 7, 14, and 21 of the starved culture, with the two-cell stage peaking at hour 20 or 24 and the four-cell stage peaking at hour 24 (Fig. 6B). Thus, the *Chlorella* endosymbionts continued to proliferate in the starved hosts at least until day 21, regardless of the presence or absence of nitrogen sources in the medium, although the proliferation rate gradually decreased (Fig. 6B). On the basis of the percentages of two-cell and four-cell stage cells, cell division of the *Chlorella* endosymbionts resulted in a 15.9 to 5.5% increase in cell number in the nitrogen-replete medium and a 7.3 to 3.4% increase in the nitrogen-depleted medium (Fig. 6C).

Then, we checked whether the *Chlorella* endosymbionts proliferating in the light are continuously digested by the host in the starved cultures. As in the starved *Paramecium* host in the dark, where the number of *Chlorella* endosymbionts per host cell gradually decreased, *Chlorella* endosymbionts being digested were also observed in the starved host under light conditions in both nitrogen-replete and nitrogen-depleted media (Fig. 1B), although the number of endosymbionts per host cell remained constant for at least 21 days (Fig. 1C). The percentage of *Chlorella* endosymbionts being digested

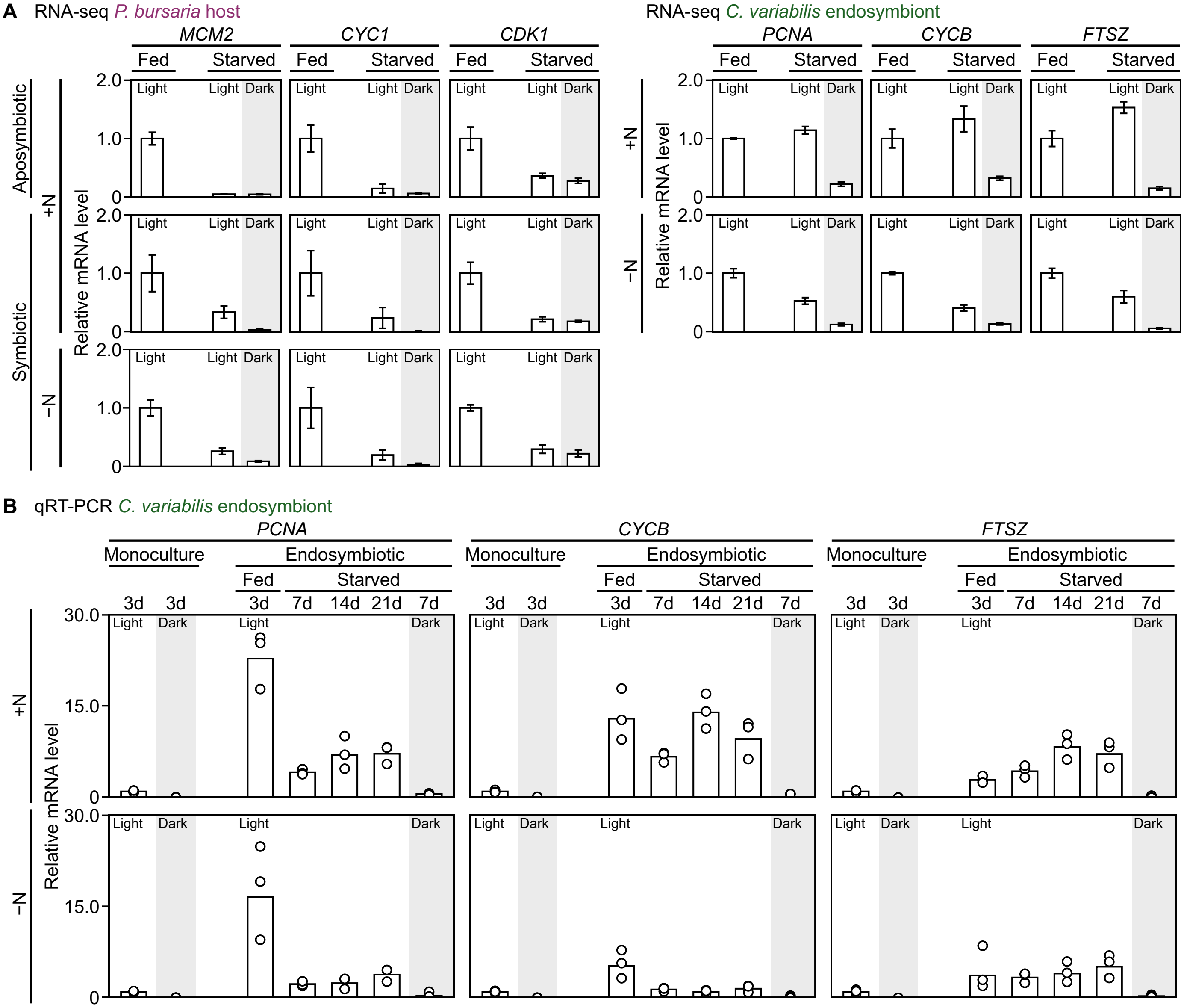

Fig. 5. Changes in the mRNA levels of S/M phase–specific genes in the *P. bursaria* host and *C. variabilis* endosymbiont under fed and starved conditions. (**A**) mRNA levels of S/M phase–specific genes in the *P. bursaria* host and *C. variabilis* endosymbiont were compared on the basis of RNA-seq results (mean ± SD; four independent cultures for each condition). Samples included aposymbiotic *P. bursaria* and *P. bursaria* cells harboring *C. variabilis* endosymbionts cultured in nitrogen-replete (mAF-6; +N) and nitrogen-depleted (mAF-6ΔN; −N) media under light or dark conditions. These were compared with samples under fed conditions in the light. For the starved condition, cells were analyzed 3 days after the initiation of starvation. The mRNA level (TPM) of each gene in the fed culture under light is defined as 1.0. (**B**) qRT-PCR results (bars indicate means; circles represent data from three independent cultures) comparing mRNA levels of S/M phase–specific genes in *C. variabilis* endosymbionts within *P. bursaria* under fed or starved conditions, in nitrogen-replete and nitrogen-depleted media, under light or dark conditions. For reference, monocultured *C. variabilis* cells in nitrogen-replete and nitrogen-depleted media under light or dark conditions were also analyzed. In monocultures, *C. variabilis* cells were analyzed 3 days (d) after the initiation of the cultures (see Fig. 2). In endosymbiotic conditions, *C. variabilis* endosymbionts within *P. bursaria* were analyzed at day 3 under fed conditions; 7, 14, and 21 days after starvation initiation under light and at day 7 under dark conditions (see Fig. 1). The mRNA level of each gene in monocultured cells under light is defined as 1.0. The IDs, read counts, and TPM values of the genes from the RNA-seq analysis are listed in datasets S1 to S3. Note that fed samples under dark conditions were not analyzed, and thus no bars are shown in the graphs for either the RNA-seq or qRT-PCR results.

within the host cell reached up to 3.8% under light conditions, which was lower than under dark conditions, where the percentage increased over time and reached up to 32.7% (fig. S6). In addition, under light conditions, starved hosts in nitrogen-replete media digested more *Chlorella* endosymbionts (up to 3.8%) than those in nitrogen-depleted media (up to 1.9%) (fig. S6).

Probable retention of iron and phosphate, as well as nitrogen, through cyclical farming in the closed *Paramecium-Chlorella* photosymbiotic system

As described above, in the starved *Paramecium* host in the nitrogen-depleted medium, *Chlorella* endosymbionts continue to proliferate in the light, and approximately the same number of endosymbionts

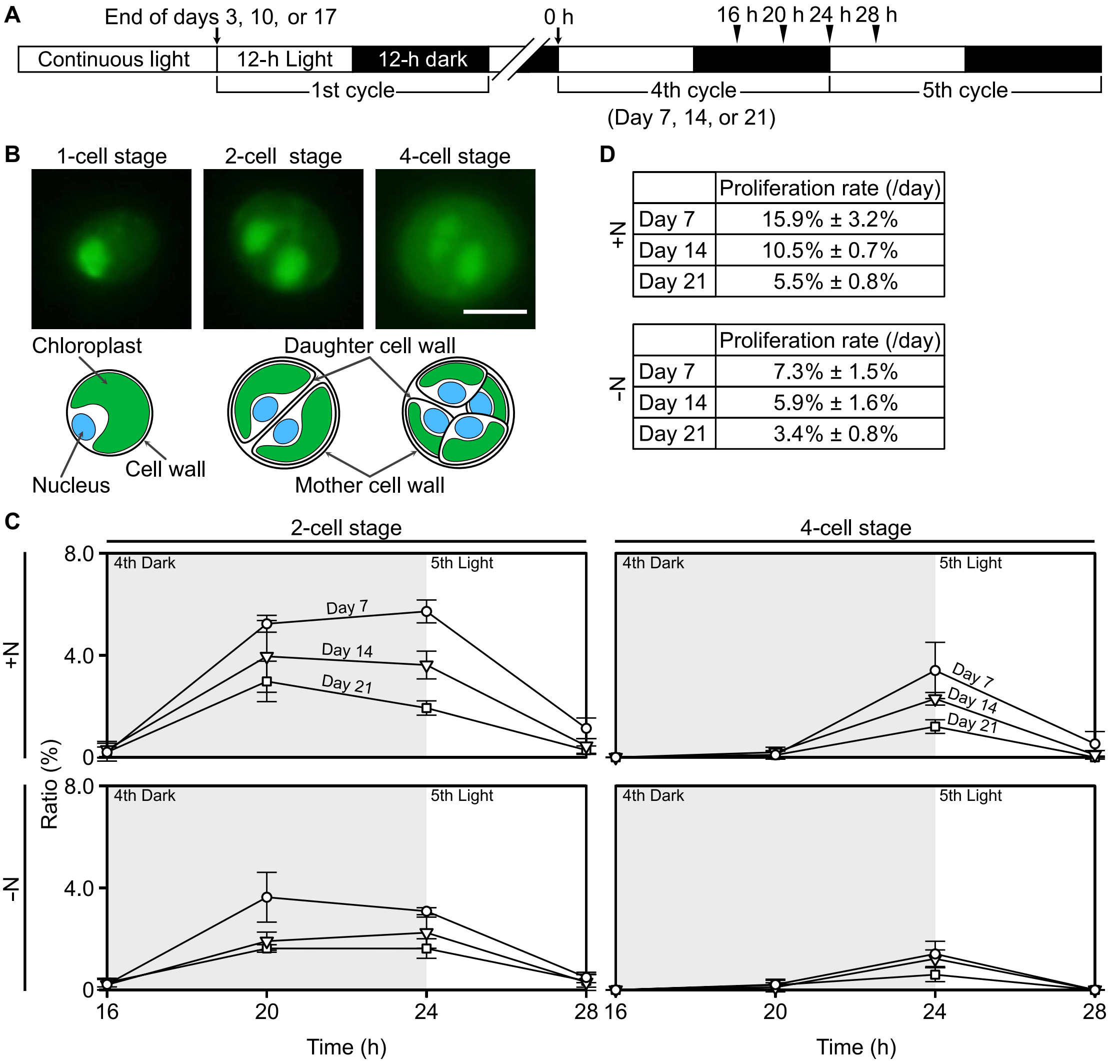

Fig. 6. Progression of the cell division cycle of *C. variabilis* endosymbionts in starved *P. bursaria* hosts under the LD cycle. (**A**) Schematic diagram of the synchronous culture and sampling time points. To synchronize the cell division cycle of *C. variabilis* endosymbionts in starved *P. bursaria* hosts, starved *P. bursaria* cells cultured under light in either nitrogen-replete or nitrogen-depleted inorganic media were subjected to a LD cycle, starting 3 days before the indicated day (corresponding to the fourth LD cycle) of the starvation culture. Cells were collected at 16, 20, and 24 hours (h; fourth cycle) and at 28 hours (i.e., 4 hours into the fifth cycle) and analyzed. (**B**) Representative fluorescence micrographs of one-cell, two-cell, and four-cell stages of *C. variabilis* cells isolated from *P. bursaria* hosts. To visualize nuclear DNA, cells were stained with GelGreen. (**C**) Changes in the percentages of two-cell and four-cell stage *C. variabilis* endosymbionts during the synchronous culture. (**D**) Daily proliferation rate of *C. variabilis* endosymbionts under each condition, estimated from the results shown in (C).

as the newly proliferated cells is consumed by the host. This observation suggests that the nitrogen sources required for the proliferation of the *Chlorella* endosymbionts are derived from metabolites generated when the *Paramecium* host digests and assimilates its *Chlorella* endosymbionts. We then asked whether this recycling of materials between the host and endosymbiont is also applicable to phosphorus and iron, which, along with nitrogen, are required for algal growth by photosynthesis and act as limiting factors in natural environments (*48*, *49*).

To this end, the *Paramecium* host harboring *Chlorella* endosymbionts was subjected to starvation in the light in an inorganic medium lacking phosphate and iron, as well as nitrogen (mAF-6ΔNPFe; −N −P −Fe) (Fig. 7). Compared to starvation cultures in normal (mAF-6) and nitrogen-depleted (mAF-6ΔN) media, the *Paramecium* hosts began to die earlier in the phosphate- and iron-depleted medium (Fig. 7A). However, nearly all of them survived for up to 3 weeks with only a slight reduction in the number of *Chlorella* endosymbionts per host cell (Fig. 7B). These results suggest that, although perhaps less efficiently than nitrogen, phosphorus and iron are also likely recycled between the host and the endosymbionts through the farming and digestion of *Chlorella* endosymbionts by the heterotrophic *Paramecium* host.

Nitrogen and phosphorus retention in the *Paramecium-Chlorella* photosymbiotic system under starvation

Next, we examined whether, as expected, nitrogen and phosphorus are retained within the closed *Paramecium-Chlorella* system without

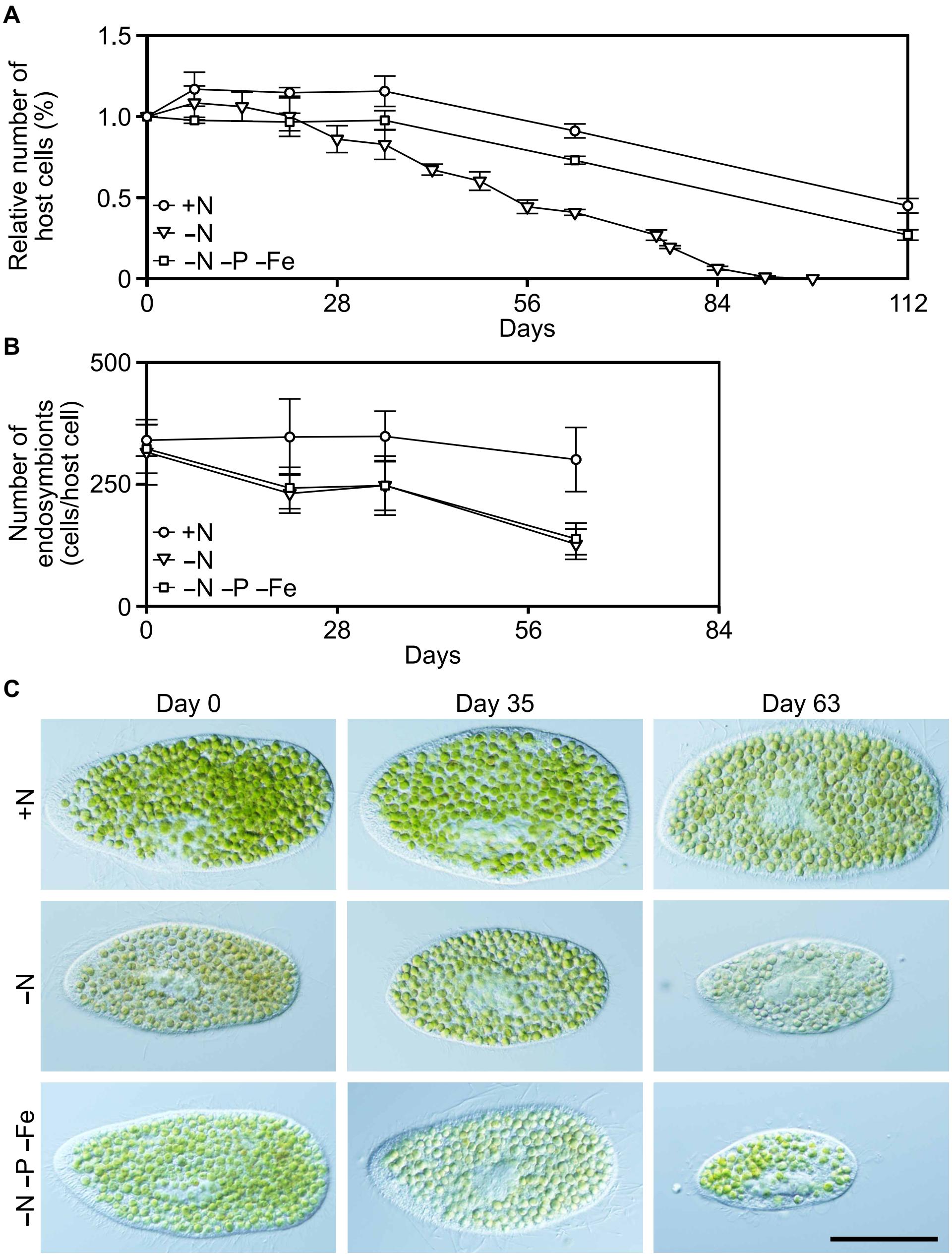

Fig. 7. Changes in the number and morphology of *P. bursaria* hosts and *C. variabilis* endosymbionts during starvation in normal, nitrogen-depleted, and nitrogen-, phosphate-, and iron-depleted inorganic media. (**A** and **B**) Changes in the number of *P. bursaria* host cells (expressed as a percentage relative to day 0; 100% corresponds to approximately 200 cells/ml in each culture) (A) and the number of *C. variabilis* endosymbionts per *P. bursaria* host cell (B) during starvation under light conditions in normal (mAF-6; +N), nitrogen-depleted (mAF-6ΔN; −N), and nitrogen-, phosphate-, and iron-depleted (mAF-6ΔNPFe; −N − P − Fe) media are shown. Means ± SD from four independent cultures are shown. (**C**) Micrographs showing *P. bursaria* cells harboring *C. variabilis* endosymbionts during starvation under light conditions in each medium. Scale bar, 50 μm.

being excreted into the medium. To this end, we measured changes in ammonium, a nitrogenous metabolite excreted by *P. bursaria* (*15*), as well as phosphate levels in the medium during starvation culture of the *Paramecium* host harboring *Chlorella* endosymbionts in the light. For comparison, we also examined these levels during starvation culture in the dark, in which the *Chlorella* endosymbionts are continuously digested but do not grow, and thus heterotrophic metabolites produced by the host cannot be reutilized by the *Chlorella* endosymbionts. To clearly observe changes in the concentrations of these substances, the starvation culture was initiated in an inorganic medium lacking phosphate and iron, as well as nitrogen (mAF-6ΔNPFe).

As expected, in the starvation culture in the dark, the ammonium level continued to increase until day 10, just before the *Paramecium* host cells began to die (Fig. 8), while in the culture under light conditions, such an increase in ammonium was not observed until day 21, just before the *Paramecium* hosts began to die (Fig. 8). Regarding phosphate, in the dark culture, its concentration increased continuously from the beginning until day 10 (Fig. 8), while in the light

culture, it remained below the detection limit until day 7, after which it became detectable and continued to increase (Fig. 8). The maximum phosphate concentration in the medium was 0.25 μM in the dark and 0.20 μM in the light, corresponding to only approximately 1/50 and 1/60, respectively, of the maximum ammonium concentration (12 μM) in the dark (Fig. 8). These results suggest that, as long as the *Paramecium* host remains alive and light energy is available, nitrogen is almost completely recycled between the host and the *Chlorella* endosymbionts, and although a small amount of phosphorus is excreted, it is also partially recycled.

Paramecium host feeds on *Chlorella* endosymbionts even while feeding on microbial prey

Last, we asked whether the *Paramecium* host feeds on *Chlorella* endosymbionts even while feeding on microbial prey. In the experiments described above, the green alga *R. fusiformis* was used as prey; however, since it is difficult to distinguish between *Chlorella* endosymbionts being digested and *R. fusiformis* prey being digested under the microscope, we used colorless *Escherichia coli* as prey in the following experiments (Fig. 9 and fig. S1).

First, we confirmed that *P. bursaria* can proliferate in an inorganic medium lacking nitrogen, phosphorus, and iron sources (mAF-6ΔNPFe) by feeding solely on *E. coli* while maintaining the number of *Chlorella* endosymbionts in its cells in the light (Fig. 9, A and B). Then, we examined starved and fed *Paramecium* hosts in the light by microscopy. Although the proportion of *Chlorella* endosymbionts being digested was higher under starvation, *Chlorella* endosymbionts being digested were also observed in *Paramecium* hosts feeding on *E. coli* (Fig. 9, A and C). These *Chlorella* cells being digested were not derived from other *Paramecium* host cells that had died in the culture medium and were ingested by the host as prey, because the experiment was conducted by culturing a single host cell in one well of a 96-well plate, and no host cell death was observed during the culture period (Fig. 9B and fig. S1).

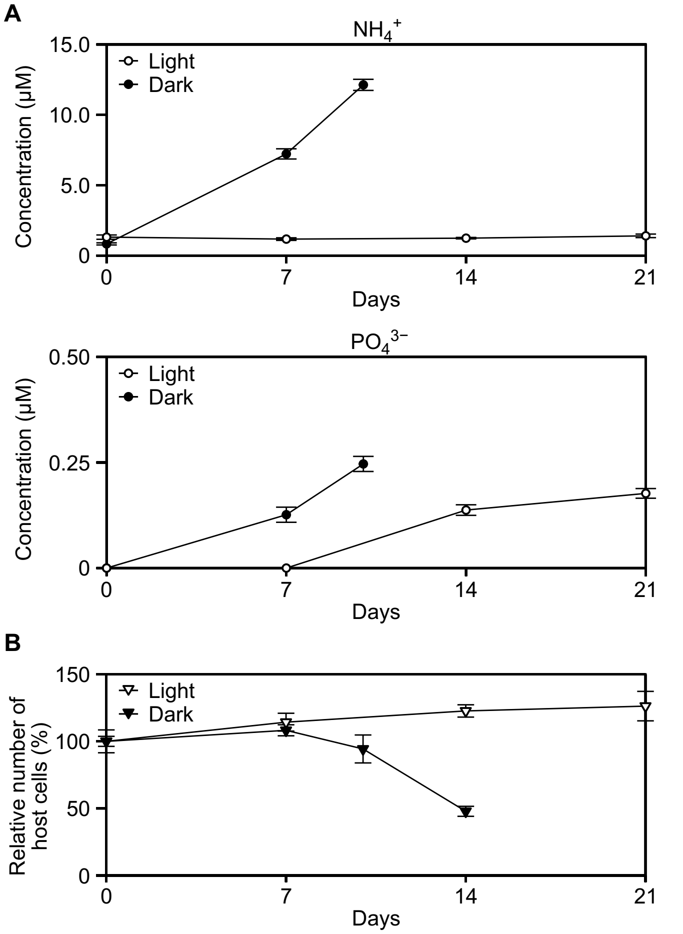

Fig. 8. Changes in ammonium and phosphate levels in the medium after *P. bursaria* cells harboring *C. variabilis* endosymbionts were subjected to starvation. *P. bursaria* cells harboring *C. variabilis* were subjected to starvation culture in nitrogen-, phosphate-, and iron-depleted medium (mAF-6ΔNPFe) under light or dark conditions. (**A**) Changes in ammonium and phosphate levels in the medium. Means ± SD from four independent cultures are shown. (**B**) Change in the *P. bursaria* cell number during the culture (expressed as a percentage relative to day 0; 100% corresponds to approximately 200 cells/ml in each culture). As in (B) and Fig. 1, nearly all *P. bursaria* cells survived up to day 10 in the dark. However, they began to die after that point, so there are no data beyond day 10 for the dark condition.

DISCUSSION

Previous studies on *P. bursaria* have shown that the *Paramecium* host digests *Chlorella* endosymbionts in specific cases. When an aposymbiotic host cell ingests *Chlorella* cells, most of the cells are digested before a portion of them escapes digestion, proliferates, and forms a stable symbiotic relationship with the host (*50*). Under prolonged dark conditions (*51*, *52*), or in the presence of cycloheximide in the light, which inhibits protein synthesis in the *Chlorella* endosymbionts, *Chlorella* endosymbionts continue to be digested by host cells, and the number of endosymbionts per host cell continues to decrease (*38*).

In this study, we have shown that a portion of *Chlorella* endosymbionts is continuously digested by the host cell, even when the host is feeding on other microbial prey and under light conditions in which the *Chlorella* endosymbionts supply photosynthates to the host. The digestion rate of endosymbionts by the host varied depending on the condition: The host digested more endosymbionts in the dark than in the light (fig. S6). The rate was also higher in starved hosts compared to those feeding on microbial prey (Fig. 9). Thus, the *Paramecium* host appears to consume more *Chlorella* endosymbionts when it lacks access to microbial food or photosynthates from the endosymbionts. In addition, the digestion rate of the *Chlorella* endosymbionts by the starved *Paramecium* host in the light was higher in the nitrogen-replete medium than in the nitrogen-depleted medium (fig. S6), presumably because the proliferation rate of *Chlorella* was also higher in the nitrogen-replete medium under light conditions (Fig. 6)

Starved *Paramecium* hosts, which survive for a long time in the light but not in the dark—previously thought to do so by relying solely on photosynthates supplied by the *Chlorella* endosymbionts (*26*)—were found to also feed on the endosymbionts, which proliferate within the host cells, thereby maintaining their number per host cell. Because the *Chlorella* endosymbionts continued to proliferate in starved hosts in media lacking nitrogen sources, as well as iron and phosphate, the existence of the following cycle is suggested: (i) The *Paramecium* host feeds on algal endosymbionts. (ii) Substances containing nitrogen, phosphate, and iron, produced through heterotrophic metabolism by the host, are supplied to the remaining

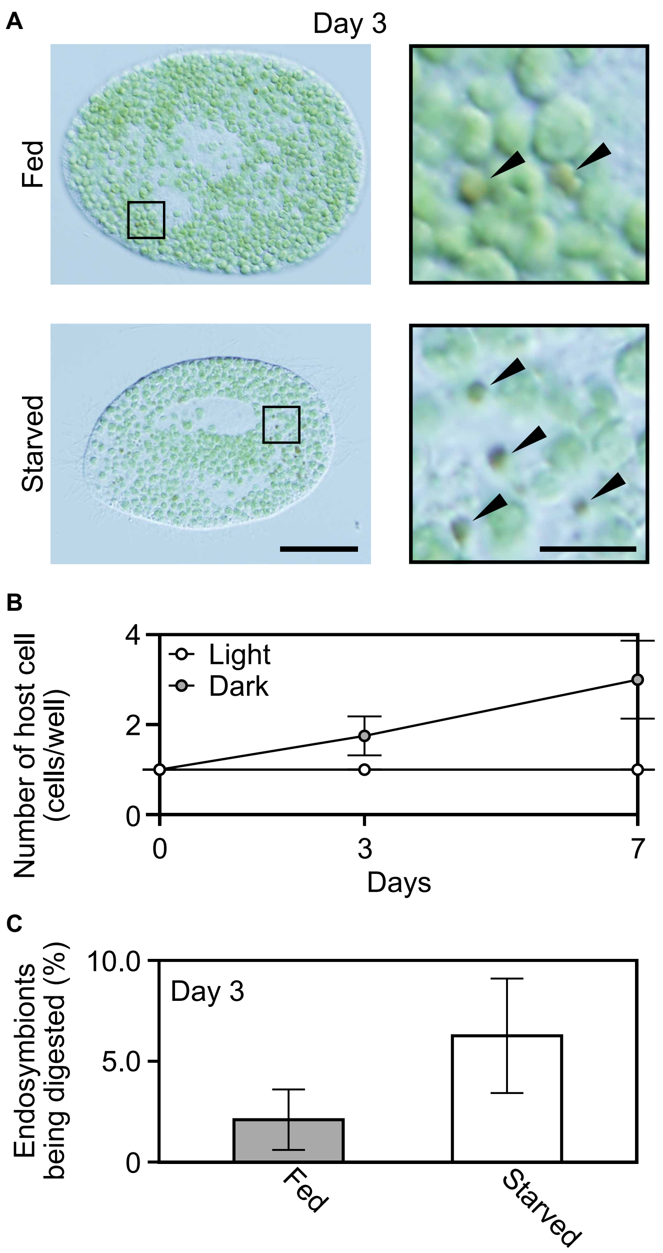

Fig. 9. Digestion of *C. variabilis* endosymbionts by *P. bursaria* hosts under fed and starved conditions. *P. bursaria* cells harboring *C. variabilis* endosymbionts were grown in nitrogen-, phosphate-, and iron-depleted medium (mAF-6ΔNPFe) supplemented with *E. coli* as prey under light conditions. A single *P. bursaria* cell was then transferred to a well of a 96-well plate containing fresh medium with (fed) or without (starved) *E. coli* prey (defined as day 0) and cultivated under light conditions. (**A**) Micrographs of *P. bursaria* cells with *C. variabilis* endosymbionts on day 3 in the fed and starved cultures (left; scale bar, 50 μm). Enlarged images of the boxed regions, in which arrowheads indicate endosymbionts being digested, are also shown (right; scale bar, 5 μm). (**B**) Changes in the number of *P. bursaria* cells per well in fed and starved cultures (mean ± SD; $n = 8$ wells for each condition). (**C**) Percentage of *C. variabilis* endosymbionts being digested on day 3 in fed and starved cultures (mean ± SD; $n = 5$ *P. bursaria* cells harboring *C. variabilis* per condition).

Chlorella endosymbionts as fertilizer. (iii) The *Chlorella* endosymbionts proliferate photosynthetically (Fig. 10). This cycle alone does not increase the total biomass of the host and endosymbionts but can prolong their longevity until the host occasionally feeds on microbial prey in prey-scarce environments, which then leads to an increase in the biomass of both the host and endosymbionts. The recycling of nutrients between the heterotrophic host and the photosynthetic endosymbiont keeps nutrient concentrations high within the closed system, preventing their dilution into the environment and reducing competition with other organisms. Thus, this system is advantageous in oligotrophic environments where free-living algae, and consequently their predators, can hardly grow.

Notably, several studies have reported the presence of bacteria associated with ciliate cells, including *P. bursaria*, and that such bacteria, for example, promote the growth of *P. bursaria (53)*. In our experiments, antibiotics were added to the culture (fig. S1) to inhibit bacterial growth; however, bacteria were not completely eliminated. Thus, it is possible that bacteria associated with *P. bursaria* may have contributed to some part of the nutrient recycling process, either by metabolizing certain compounds and/or by being digested along with *Chlorella* endosymbionts by the *Paramecium* host.

On the basis of the above conclusion, we can reasonably interpret the transcriptome results. Differences between fed and starved conditions in the light were much smaller in both the *Paramecium* host and *Chlorella* endosymbiont than those between light and dark conditions or between nitrogen-replete and nitrogen-depleted media (Figs. 3 and 4). This is probably because, even under starvation, the host feeds on endosymbionts and supplies them with heterotrophic metabolites, as in the fed condition. In the host t-SNE plot (Fig. 3A), the bottom right group lacks any food source (neither prey nor endosymbionts); the upper right group feeds only on prey; and the left group feeds on endosymbionts or both. In the endosymbiont t-SNE plot, the bottom right group neither utilizes nitrogen sources nor grows due to the absence of light energy. The top center group uses host-derived metabolites as nitrogen sources. The upper left group uses both host metabolites and inorganic nitrogen in the medium.

It should be noted that, in *P. bursaria*, the above-mentioned cyclical farming does not function perfectly without any loss. A previous study showed that, after daughter *Chlorella* endosymbionts hatch out from the mother cell, the mother cell wall is transferred from the symbiosome to the digestive vacuole and is excreted outside the host cell (*35*). In our study, during the later stage of starvation culture of *Paramecium* hosts harboring the *Chlorella* endosymbionts in the light, we observed the accumulation of debris excreted by the host cells at the bottom of the culture flasks, including indigestible parts of the cell walls of *Chlorella* endosymbionts (fig. S7). Thus, the *Paramecium* host appears to be unable to digest and absorb all of the biomass of the *Chlorella* endosymbionts. Despite this incompleteness, the numbers of both the host and endosymbionts were almost maintained for up to 5 weeks in the nitrogen-depleted medium, and up to 3 weeks in the nitrogen-, phosphorus-, and iron-depleted medium without prey, before eventually decreasing.

Another important point is that expulsion of intact endosymbionts—a known mechanism for regulating symbiont populations in other photosymbiotic systems (e.g., corals) (*17*) —was not observed during starvation culture in this study, unlike the expulsion of debris derived from digested symbionts. This conclusion is based on the following observations. Throughout the study, *P. bursaria* was cultured under static conditions. If intact *Chlorella* endosymbionts

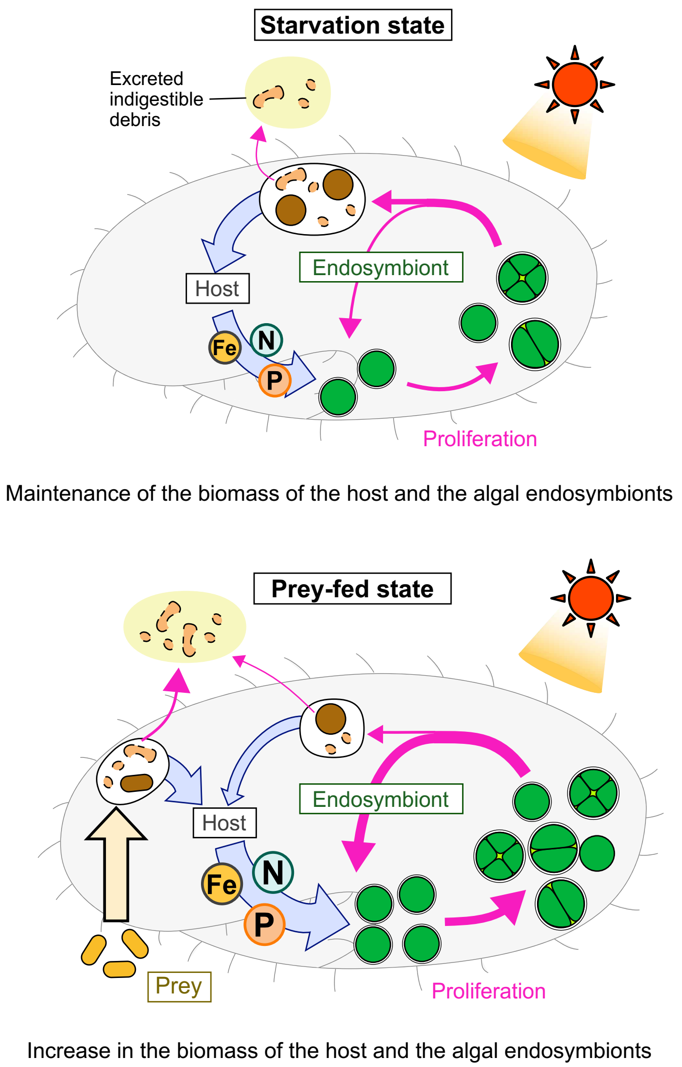

Fig. 10. Schematic representation of the cyclical farming of photosynthetic endosymbionts by the heterotrophic host in *P. bursaria*. *P. bursaria* feeds on *C. variabilis* endosymbionts. In the light, metabolites containing elements required for photosynthetic growth—such as nitrogen, phosphorus, and iron—are not excreted but instead supplied to the *C. variabilis* endosymbionts, allowing them to proliferate. This recycling cycle extends the longevity of the host and maintains the number of endosymbionts per host cell, even in the absence of microbial prey (**top**). When microbial prey is available, the host grows and proliferates, and the additional metabolites produced from prey consumption are also supplied to the endosymbionts, leading to an increase in their total biomass (**bottom**).

had been expelled, then they would likely have accumulated at the bottom of the culture flasks. However, only indigestible debris derived from digested endosymbionts was observed to accumulate (fig. S7). Notably, at the time of observation, almost all *P. bursaria* cells were still alive, and thus release of *Chlorella* endosymbionts from dead host cells was not expected. In addition, the starvation experiment in Fig. 9 was initiated with a single *P. bursaria* cell per well in a 96-well plate, to eliminate the possibility that *Chlorella* endosymbionts released from dead host cells could influence other living host cells. All cells survived the 7-day observation period, and no free intact *Chlorella* cells were detected outside the host cells.

Regarding the retention of nutrients between the host and endosymbiont, nitrogen was well retained in the starved culture under light conditions, whereas phosphorus, although present in smaller amounts by comparison, was partially excreted (Fig. 8). The biomass molecular formula of microalgae is estimated to be approximately $CH_{1.7}O_{0.4}N_{0.15}P_{0.0094}$ (*54*), indicating that phosphorus is required at only about 1/16 of the amount of nitrogen. Although it was not measurable in this study due to its trace concentration, the iron content is even lower, estimated to be about 1/3000 of nitrogen (*55*). Thus, the photoendosymbiotic relationship between the *Paramecium* host and the *Chlorella* endosymbionts may have evolved to retain nitrogen more effectively than phosphorus. Nonetheless, in the starved culture under light conditions, the longevity of the *Paramecium* host was shorter in the medium lacking nitrogen, phosphorus, and iron than in the medium lacking only nitrogen (Fig. 7). To better understand this point, future studies should investigate the water chemistry of natural habitats—specifically, which among nitrogen, phosphorus, or iron is the primary limiting factor—and incorporate those findings into further analyses.

The above conclusion raises the question of why the host, especially under starved conditions, needs to obtain energy for survival not only by receiving photosynthesized carbohydrates such as maltose from intact algal endosymbionts but also by consuming the entire algal endosymbionts. Theoretically, as previously assumed (*26*), the starved heterotrophic host might be able to obtain sufficient energy for survival through respiration using only photosynthesized carbohydrates supplied by the algal endosymbionts. However, when considering the metabolic constraints of free-living algae (*56*) and our results from the monoculture of the *Chlorella* endosymbiont (Fig. 2 and fig. S3), it is unlikely that high activity of photosynthetic carbon fixation can be sustained without cellular growth accompanied by the assimilation of other materials such as nitrogen, which leads to the production of amino acids and nucleic acids. Thus, to sustain carbon fixation, the host needs to supply nutrients, including nitrogen sources, to the algal endosymbionts and allow them to continue growing. In oligotrophic environments where microbial prey or other nitrogen sources cannot be sufficiently obtained, the host needs to feed on the endosymbionts themselves to generate heterotrophic metabolites that supply nitrogen sources to the remaining endosymbionts.

Feeding on photosynthetic or chemosynthetic endosymbionts by eukaryotic hosts has also been observed in other endosymbiotic relationships (*25*, *57*, *58*). In the case of corals, the cnidarian hosts digest or excrete dinoflagellate algal endosymbionts, and it has long been believed that such digestion or excretion serves to maintain the population balance between the host cells and the endosymbionts. However, a recent study showed that the cnidarian host obtains nitrogen and phosphorus by feeding on the algal endosymbionts, which assimilate these nutrients from the environment through their photosynthetic activity (*25*). As observed in *P. bursaria* in this study, 1 to 6% (depending on the species) of dinoflagellate endosymbionts in corals are digested per day—a value that is similar to the endosymbiont division rate (*17*, *25*, *59*). In the case of corals, a point that differs from the results of *P. bursaria* in this study is that, in water depleted of nitrogen and phosphorus sources, the host ultimately consumes all of the algal endosymbionts for growth, resulting in bleaching even under light conditions (*25*).

In addition to corals, the digestion of algal endosymbionts has also been observed in some planktonic foraminifera (*60*, *61*). Although

the significance is unclear, it is hypothesized that the host consumes the endosymbionts as a source of nutrients when external food is unavailable during the growth phase. Moreover, the algal endosymbionts are completely consumed just before gametogenesis, at which stage the host loses its feeding apparatus (spines), and thus the endosymbionts likely serve as a final nutrient reserve (*57*). As an additional example—although not a case of photosymbiosis—a recent study showed that mussels living near deep-sea vents, which harbor chemosynthetic bacterial endosymbionts, digest these symbionts when the nutrient supply from them is reduced, to compensate for the deficiency (*62*, *63*). In this regard, in the present study, starved *P. bursaria* harboring *Chlorella* endosymbionts in the dark survived longer than their aposymbiotic counterparts by consuming the endosymbionts (Fig. 1). Thus, the advantage of endosymbionts serving as emergency food reserves for the host may be common across various endosymbiotic relationships. In addition, cyclical farming and feeding on endosymbionts is also likely a widespread phenomenon in nutrient-limited environments, although it has been overlooked because, as observed in this study, the number of algal endosymbionts per host cell appears to remain constant under certain conditions, such as in the light.

MATERIALS AND METHODS

Strains and culture condition

P. bursaria (NIES-2891) was obtained from the National Institute for Environmental Studies (NIES), in which a clone (clone A1) of *C. variabilis* isolated from the original *P. bursaria* strain was reinfected into the *Paramecium* host. *P. bursaria* and monoculture of *C. variabilis* A1 were maintained in mAF-6 (*64*) medium. mAF-6 was designed as an inorganic version of the original medium, in which Fe-citrate ($FeC_6H_5O_7$) was replaced with Fe(III)-EDTA ($C_{10}H_{12}FeN_2NaO_8{\cdot}3H_2O$). mAF-6ΔN was designed as a nitrogen-depleted version of mAF-6, which does not contain sodium nitrate ($NaNO_3$) and ammonium nitrate (NH_4NO_3), but the same concentration of sodium ion (Na^+) was compensated with sodium chloride (NaCl). mAF-6ΔNPFe was designed as a nitrogen-, phosphate-, and iron-depleted version, which does not contain Fe(III)-EDTA and ferric chloride ($FeCl_3$), dipotassium hydrogenphosphate (K_2HPO_4), and potassium dihydrogen phosphate (KH_2PO_4), but the same concentration of potassium ion (K^+) was compensated with potassium chloride (KCl). All media contained MES (0.40 g/liter; $C_6H_{13}NO_4S{\cdot}H_2O$) as a buffer according to the recipe provided by NIES and were adjusted to pH 6.6 using sodium hydroxide (NaOH).

Both *P. bursaria* and *C. variabilis* were cultured in 250 ml of respective medium in a 75-cm^2 tissue culture flask (353136, Corning Inc.) at 20°C with a light intensity of 10 μmol photons m^{-2} s^{-1} provided by a fluorescent lamp (FL15N, Toshiba). To prevent precipitation, *C. variabilis* was cultured on a rotary shaker (Shaker-LR, TAITEC Corp.) at 140 rpm.

Feeding conditions for *P. bursaria*

R. fusiformis (NIES-123) was obtained from NIES as prey for *P. bursaria*. *R. fusiformis* was cultured in 250 ml of mAF-6 in a 75-cm^2 tissue culture flask at 20°C with illumination (30 μmol photons m^{-2} s^{-1}) on a rotary shaker at 140 rpm.

Before adding *R. fusiformis* as prey to the *P. bursaria* culture, the prey culture was centrifuged at 4300*g* for 10 min using a 50-ml tube. The cell pellet was washed twice by resuspending it in a medium for *P. bursaria* culture and was lastly resuspended in the medium to give an optical density at 750 (OD_{750}) = 5.0. 1 ml of the washed *R. fusiformis* suspension added to 250 ml of *P. bursaria* culture. *P. bursaria* was fed two to three times a week, once the previously provided prey had been consumed.

Preparation of aposymbiotic *P. bursaria*

To prepare aposymbiotic *P. bursaria*, *P. bursaria* harboring *C. variabilis* endosymbionts were cultured for ~2 weeks in mAF-6 supplemented with cycloheximide (10 μg/ml) in the light, according to a previous report (*38*). After the treatment, the complete loss of *C. variabilis* endosymbionts in *P. bursaria* hosts was confirmed under a fluorescent microscope by observing chlorophyll fluorescence. A portion of the aposymbiotic *P. bursaria* cells was then transferred and cultured in mAF-6 medium with *R. fusiformis* as prey under light conditions, as described above.

Starvation culture of *P. bursaria*

Before exposing *P. bursaria* to starvation, *P. bursaria* was given a surplus of prey and cultured for 3 days. Then, to remove free prey that had not been ingested by *P. bursaria* from the medium, the culture was filtered through a 5-μm pore nylon mesh (NY5-HC, Sefar AG), and *P. bursaria* cells on the mesh were washed with fresh medium and then resuspended in a fresh medium without prey. In addition, carbenicillin and streptomycin [water stock (50 mg/ml)] were added to give a final concentration of 50 μg/ml for each after the free prey removal step to prevent contamination by bacteria, which could serve as a food source for *P. bursaria*. Subsequently, *P. bursaria* cells were cultured without prey for an additional 2 days to allow complete digestion of the ingested prey. Last, *P. bursaria* cells were filtered and washed as described above to remove materials excreted by *P. bursaria* during prey digestion and then adjusted to approximately 200 *Paramecium* cells/ml in fresh medium containing the antibiotics described above. To track changes in the cell numbers of *P. bursaria* and *C. variabilis* endosymbionts, 40 ml of the culture was dispensed into a 25-cm^2 flask, while for RNA sampling and quantification of ammonium and phosphate in the culture medium, 250 ml of the culture was dispensed into a 75-cm^2 flask. Here, we defined this time point as day 0, and then the *P. bursaria* was cultured as described above, except that the flask was covered with aluminum foil for the dark condition. For comparison in some analyses, a fed culture control was prepared by adding *R. fusiformis* prey, as described above, to the day 0 culture (the procedures described above are illustrated in fig. S1).

To examine the cell number of *P. bursaria*, a 0.5-ml culture was fixed with 1% glutaraldehyde, and the total cell count was determined under an inverted microscope (CKX53, Olympus) equipped with 4× objective lens at the indicated time point. Only living cells, distinguished by a smooth outline and a clear border, unlike dead cells, were included in the count. Furthermore, cell details were observed using a microscope (BX51, Olympus) equipped with differential interference optics and 40× objective lenses. The experiments involving the host *P. bursaria* were performed in four biological replicates ($n = 4$) for each condition. For the *C. variabilis* endosymbiont, five host cells containing endosymbionts were used as biological replicates ($n = 5$) for each condition.

To synchronize the progression of the cell division cycle of *C. variabilis* endosymbiont cells accommodated in *P. bursaria* host cells, 40 ml of culture was transferred to a 25-cm^2 flask and cultivated

under the LD cycle. Five milliliters of the culture was collected at 16 and 20 hours after the start of the fourth light period and at 0 and 4 hours after the start of the fifth light period. Then, 250 μl of 2% SDS solution was added and vortexed to dissolve the *P. bursaria* host cells but not the *C. variabilis* endosymbiont cells due to the presence of a cell wall. Cells were fixed with 4% paraformaldehyde, and the nucleus was stained with 0.02% GelGreen (Biotium Inc.) before being observed using a fluorescent microscope (BX51, Olympus). This experiment was performed in four biological replicates ($n = 4$) in each condition.

C. variabilis monoculture in a nitrogen-replete or nitrogen-depleted inorganic medium

C. variabilis A1 cells in the stock culture maintained in mAF-6 medium were centrifuged at 4300*g* for 10 min and washed twice with mAF-6 or mAF-6ΔN medium. The cells were inoculated into 200 ml of the respective medium in a 75-cm^2 flask to give a density of $OD_{750} = 0.1$ and then cultured at 20°C in the light (10 μmol photons m^{-2} s^{-1}) on a rotary shaker at 140 rpm. After 1 week of cultivation, cells cultured in the light were harvested by centrifugation at 4300*g* for 10 min and resuspended in 200 ml of the respective fresh medium to give a density of $OD_{750} = 0.1$, again being cultivated as above. The cell number was counted using a hemacytometer at the indicated time points. All experiments were performed in four biological replicates ($n = 4$) in each condition.

Chlorophyll quantification

For quantification of chlorophyll *a* and *b*, *P. bursaria* cells harboring *C. variabilis* endosymbionts or monocultured *C. variabilis* cells were collected from 5 ml of culture by centrifugation at 4300*g* for 10 min. To prevent the cells from adhering to the walls of the centrifuge tube, Tween 20 was added to a final concentration of 0.02% just before centrifugation. The pellet was resuspended in 1 ml of *N,N*-dimethylformamide to extract chlorophyll *a* and *b*. After extraction, the cells were removed by centrifugation at 4300*g* for 10 min. The absorbance of the supernatant at 647 and 664 nm was measured using a spectrophotometer. Chlorophyll *a* and *b* contents were calculated as described (*65*). All experiments were performed in four biological replicates ($n = 4$) in each condition.

qRT-PCR and RNA-seq analyses

To extract the total RNA, the cells were harvested from 50 ml of the respective cultures by centrifugation at 4300*g* for 7 min. To prevent the cells from adhering to the walls of the centrifuge tube, 50 μl of 20% Tween 20 was added to the culture just before centrifugation. The cell pellets were immediately frozen in liquid nitrogen and then stored at −80°C until use. Total RNA was extracted using a phenol/chloroform method (*66*), and 1.3 to 17.6 μg of total RNA was used for qRT-PCR and RNA-seq analyses.

For qRT-PCR, 0.25 μg of total RNA was reverse-transcribed into cDNA using PrimeScript reverse transcriptase (Takara) with a random hexamer, following the manufacturer's instructions, and a 1/40 aliquot of cDNA was used for each qPCR reaction. qPCR was performed using the CFX Duet Real-Time PCR System (Bio-Rad) and SYBR Green qPCR Master Mix (Thermo Fisher Scientific Inc.), following the standard cycling conditions according to the manufacturer's instructions, with the annealing/extension step modified to 68°C. The primer sets, listed in dataset S8, were designed on the basis of the *C. variabilis* A1 genome sequence (BioProject accession no. PRJDB19703, Dryad doi:10.5061/dryad.zkh1893nm) and 18*S* ribosomal RNA (rRNA) sequence of *C. variabilis* NC64A. The expression values of the respective genes were normalized to the values of 18*S* rRNA as an internal control. qRT-PCR experiments were performed in three biological replicates ($n = 3$) in each condition.

For RNA-seq, the RNA samples were subjected to library construction with the NEBNext Ultra RNA LP Kit (New England Biolabs) and 150-bp paired-end, strand-specific sequencing by NovaSeq 6000. The adapter sequence was removed using Fastp (with option --detect_adapter_for_pe, ver. 0.23.4) (*67*). In addition, reads mapped to the *C. variabilis* endosymbiont were also removed by HISAT2 (described below) for the de novo assembly of *P. bursaria* (*68*). De novo assembly for *P. bursaria* RNA-seq reads was carried out using Trinity (with option --SS_lib_type RF and --min_contig_length 500, ver. 2.15.2), clustering was done using CD-HIT (with parameter -c 0.95, ver. 4.8.1), open reading frame extraction and prediction were performed using TransDecoder (with option -G Ciliate, -m 100 and --retain_pfam_hits, ver. 5.7.1, http://transdecoder.github.io) and HMMER (with option -E 1e-5, ver. 3.4), using the Pfam-A database (March 2024) (*69*–*72*). Mapping and counting were conducted using RSEM (--hisat2-hca option for preparing index and --paired-end and --strandedness reverse for counting, ver. 1.3.3) with HISAT2 (ver. 2.2.1). Annotation was performed using DIAMOND in blastp mode (with options --evalue 1e-5 and --ultra-sensitive, ver. 2.1.11), using the National Center for Biotechnology Information non-redundant (nr) database (November 2023) (*68*, *73*, *74*). Assembled sequences that appeared to originate from artificial sources, viruses, bacteria, archaea, Viridiplantae, Rhodophyta, and fungi were removed to obtain RNA-seq results specific to *P. bursaria*. KEGG annotation was performed using the KEGG Automatic Annotation Server (version 2.1) with amino acid sequences (*75*). In addition, sequences with transcripts per million (TPM) values below 1 were also removed. For the analysis of *C. variabilis* RNA-seq reads, the reference of *C. variabilis* genome (BioProject accession no. PRJDB19703) was used. The output of raw read counts from RSEM was used for t-SNE, which was performed in R (ver. 4.3.2) using Rtsne (with options pca = True, perplexity = 5, theta = 0, and iteration = 10,000, ver. 0.17), and differential gene expression analysis using edgeR (ver. 4.0.16) with an FDR threshold of 0.05 and a $\log_2$ fold change greater than 1 or less than −1 (*76*–*78*). RNA-seq was performed in four biological replicates ($n = 4$) in each condition.

Observation of *P. bursaria* hosts digesting *C. variabilis* endosymbionts in a prey-supplemented medium

P. bursaria was fed *E. coli* instead of *R. fusiformis* because both are unicellular green algae, making it difficult to distinguish between *C. variabilis* and *R. fusiformis* while they were being digested under a microscope. *E. coli* was cultivated in 5 ml of LB medium at 37°C on a shaker (DWMax V BR-104, TAITEC Corp.) at 270 rpm overnight. *E. coli* was then washed with mAF-6ΔNPFe and was lastly resuspended in 250 ml of *P. bursaria* culture at a final density of $OD_{600} = 0.3$, twice a week, once the previously provided prey had been consumed.

To observe the *P. bursaria* host cell digesting *C. variabilis* endosymbionts under fed and starved conditions, *P. bursaria* cells cultured with *E. coli* prey were washed by pipetting on a blood cell reaction plate with fresh mAF-6ΔNPFe medium. After nine washes, the single cells were transferred individually to a well of a 96-well plate.

The fed group received 200 μl of mAF-6ΔNPFe medium (without any antibiotics) with *E. coli* at an OD_{600} of 0.15, while the starved group did not receive any food. The cell number of *P. bursaria* in each

well was counted at the indicated time points. *C. variabilis* endosymbionts being digested in the *P. bursaria* host cell were observed using a microscope (BX51, Olympus) equipped with differential interference optics and 40× objective lenses. The experiments involving the host *P. bursaria* were performed in eight biological replicates ($n = 8$) for each condition. For the endosymbiont, five host cells containing endosymbionts were used as biological replicates ($n = 5$) for each condition.

Quantification of ammonium and phosphate in culture medium

To quantify ammonium and phosphate in the culture medium, 50 ml of the starved *P. bursaria* culture in mAF-6ΔNPFe medium was centrifuged at 4300*g* for 7 min to pellet the cells. To prevent cell adhesion to the walls of the centrifuge tube, 50 μl of 20% Tween 20 was added to the culture just before centrifugation. Then, 40 ml of the supernatant was collected and stored at −80°C until analysis. Ammonium and phosphate concentrations were determined using the indophenol blue and molybdenum blue colorimetric methods, respectively. All reactions were carried out in 15-ml tubes. Before phosphate measurement, 6.0 mg of activated carbon (Norit SX Plus) and 150 μl of 1 M NaOH were added to 2.85 ml of sample, followed by shaking for 2 hours at 60 rpm using a shaker (NR-20, TAITEC Corp.) to remove substances that inhibit the colorimetric reaction. After this treatment, the samples were centrifuged at 2000*g* for 10 min to remove the activated carbon, and 2.0 ml of the resulting supernatant was used for the colorimetric assay. Last, absorbance of the 2.0-ml sample was measured using a UV-visible spectrophotometer (UV-2600, Shimadzu Corp.) and compared with standards. The detection limit was 0.55 μM (10 μg/liter as NH_4^+) for ammonium and 0.11 μM (10 μg/liter as PO_4^{3-}) for phosphate. All data below the detection limit were treated as 0 in the analysis. All experiments were performed in four biological replicates ($n = 4$).

Supplementary Materials

The PDF file includes:
Figs. S1 to S7
Legends for data S1 to S8

Other Supplementary Material for this manuscript includes the following:
Data S1 to S8

REFERENCES AND NOTES

1. R. G. Dorrell, C. J. Howe, What makes a chloroplast? reconstructing the establishment of photosynthetic symbioses. *J. Cell Sci.* **125**, 1865–1875 (2012).
2. R. A. Foster, J. P. Zehr, Diversity, genomics, and distribution of phytoplankton-Cyanobacterium single-cell symbiotic associations. *Annu. Rev. Microbiol.* **73**, 435–456 (2019).
3. S. Miyagishima, Taming the perils of photosynthesis by eukaryotes: Constraints on endosymbiotic evolution in aquatic ecosystems. *Commun. Biol.* **6**, 1150 (2023).
4. H. Takagi, K. Kimoto, T. Fujiki, H. Saito, C. Schmidt, M. Kucera, K. Moriya, Characterizing photosymbiosis in modern planktonic foraminifera. *Biogeosciences* **16**, 3377–3396 (2019).
5. M. Hamada, K. Schröder, J. Bathia, U. Kürn, S. Fraune, M. Khalturina, K. Khalturin, C. Shinzato, N. Satoh, T. C. Bosch, Metabolic co-dependence drives the evolutionarily ancient *Hydra–Chlorella* symbiosis. *eLife* **7**, e35122 (2018).
6. M. Mies, P. Y. G. Sumida, N. Rädecker, C. R. Voolstra, Marine invertebrate larvae associated with *Symbiodinium*: A mutualism from the start? *Front. Ecol. Evol.* **5**, 2699–2717 (2017).
7. B. Marin, E. C. M. Nowack, M. Melkonian, A plastid in the making: Evidence for a second primary endosymbiosis. *Protist* **156**, 425–432 (2005).
8. S. J. Sibbald, J. M. Archibald, Genomic insights into plastid evolution. *Genome Biol. Evol.* **12**, 978–990 (2020).
9. J. Gentil, F. Hempel, D. Moog, S. Zauner, U. G. Maier, Review: Origin of complex algae by secondary endosymbiosis: A journey through time. *Protoplasma* **254**, 1835–1843 (2017).
10. A. A. Venn, J. E. Loram, A. E. Douglas, Photosynthetic symbioses in animals. *J. Exp. Bot.* **59**, 1069–1080 (2008).
11. Y. Kato, S. Ueno, N. Imamura, Studies on the nitrogen utilization of endosymbiotic algae isolated from Japanese *Paramecium bursaria*. *Plant Sci.* **170**, 481–486 (2006).
12. M. He, J. Wang, X. Fan, X. Liu, W. Shi, N. Huang, F. Zhao, M. Miao, Genetic basis for the establishment of endosymbiosis in *Paramecium*. *ISME J.* **13**, 1360–1369 (2019).
13. W. Reisser, The metabolic interactions between *Paramecium bursaria* Ehrbg. and *Chlorella* spec. in the *Paramecium bursaria*-symbiosis. *Arch. Microbiol.* **125**, 291–293 (1980).
14. E. Ziesenisz, W. Reisser, W. Wiessner, Evidence of de novo synthesis of maltose excreted by the endosymbiotic *Chlorella* from *Paramecium bursaria*. *Planta* **153**, 481–485 (1981).
15. D. Albers, W. Reisser, W. Wiessner, Studies on the nitrogen supply of endosymbiotic chlorellae in greem *Paramecium bursaria*. *Plant Sci. Lett.* **25**, 85–90 (1982).
16. M. E. S. Sørensen, A. J. Wood, E. J. A. Minter, C. D. Lowe, D. D. Cameron, M. A. Brockhurst, Comparison of independent evolutionary origins reveals both convergence and divergence in the metabolic mechanisms of symbiosis. *Curr. Biol.* **30**, 328–334.e4 (2020).
17. S. K. Davy, D. Allemand, V. M. Weis, Cell biology of Cnidarian-Dinoflagellate symbiosis. *Microbiol. Mol. Biol. Rev.* **76**, 229–261 (2012).
18. R. K. Trench, The physiology and biochemistry of zooxanthellae symbiotic with marine coelenterates III. The effect of homogenates of host tissues on the excretion of photosynthetic products in vitro by zooxanthellae from two marine coelenterates. *Proc. R. Soc. Lond. B Biol. Sci.* **177**, 251–264 (1971).
19. S. G. Leles, A. Mitra, K. J. Flynn, D. K. Stoecker, P. J. Hansen, A. Calbet, G. B. McManus, R. W. Sanders, D. A. Caron, F. Not, G. M. Hallegraeff, P. Pitta, J. A. Raven, M. D. Johnson, P. M. Glibert, S. Våge, Oceanic protists with different forms of acquired phototrophy display contrasting biogeographies and abundance. *Proc. R. Soc. B or Proc. Biol. Sci.* **284**, 20170664 (2017).
20. S. Woelfl, P. Garcia, C. Duarte, Chlorella-bearing ciliates (*Stentor*, *Ophrydium*) dominate in an oligotrophic, deep North Patagonian lake (Lake Caburgua, Chile). *Limnologica* **40**, 134–139 (2010).
21. T. M. Sonneborn, "Methods in *Paramecium* research" in *Methods in Cell Biology* (Academic Press, 1970), vol. 4, pp. 241–339, 10.1016/S0091-679X(08)61758-6.
22. S. G. Berk, L. H. Parks, R. S. Ting, Photoadaptation alters the ingestion rate of *Paramecium bursaria*, a mixotrophic ciliate. *Appl. Environ. Microbiol.* **57**, 2312–2316 (1991).
23. H. M. Lenhoff, R. D. Brown, Mass culture of hydra: An improved method and its application to other aquatic invertebrates. *Lab. Anim* **4**, 139–154 (1970).
24. Y.-L. Huang, A. B. Mayfield, T.-Y. Fan, Effects of feeding on the physiological performance of the stony coral *Pocillopora acuta*. *Sci. Rep.* **10**, 19988 (2020).
25. J. Wiedenmann, C. D'Angelo, M. L. Mardones, S. Moore, C. E. Benkwitt, N. A. J. Graham, B. Hambach, P. A. Wilson, J. Vanstone, G. Eyal, O. Ben-Zvi, Y. Loya, A. Genin, Reef-building corals farm and feed on their photosynthetic symbionts. *Nature* **620**, 1018–1024 (2023).
26. S. J. Karakashian, Growth of *Paramecium bursaria* as influenced by the presence of algal symbionts. *Physiol. Zool.* **36**, 52–68 (1963).
27. G. Omura, M. Ishida, M. Arikawa, S. M. M. K. Khan, Y. Suetomo, S. Kakuta, C. Yoshimura, T. Suzaki, A bacteria-free monoxenic culture of *Paramecium bursaria*: Its growth characteristics and the re-establishment of symbiosis with *Chlorella* in bacteria-free conditions. *Jpn. J. Protozool.* **37**, 139–150 (2004).
28. E. L. Horas, S. M. Metzger, B. Platzer, J. B. Kelly, L. Becks, Context-dependent costs and benefits of endosymbiotic interactions in a ciliate–algae system. *Environ. Microbiol.* **24**, 5924–5935 (2022).
29. S. Iwai, K. Fujita, Y. Takanishi, K. Fukushi, Photosynthetic endosymbionts benefit from host's phagotrophy, including predation on potential competitors. *Curr. Biol.* **29**, 3114–3119.e3 (2019).
30. T. Tyrrell, The relative influences of nitrogen and phosphorus on oceanic primary production. *Nature* **400**, 525–531 (1999).
31. W. Koerselman, A. F. M. Meuleman, The vegetation N:P ratio: A new tool to detect the nature of nutrient limitation. *J. Appl. Ecol.* **33**, 1441 (1996).
32. J. H. Martin, Glacial-interglacial CO_2 change: The iron hypothesis. *Paleoceanography* **5**, 1–13 (1990).
33. R. L. North, S. J. Guildford, R. E. H. Smith, S. M. Havens, M. R. Twiss, Evidence for phosphorus, nitrogen, and iron colimitation of phytoplankton communities in Lake Erie. *Limnol. Oceanogr.* **52**, 315–328 (2007).
34. C. Dziallas, M. Allgaier, M. T. Monaghan, H.-P. Grossart, Act together—Implications of symbioses in aquatic ciliates. *Front. Microbiol.* **3**, 288 (2012).
35. Y. Kodama, M. Fujishima, Cell division and density of symbiotic *Chlorella variabilis* of the ciliate *Paramecium bursaria* is controlled by the host's nutritional conditions during early infection process. *Environ. Microbiol.* **14**, 2800–2811 (2012).
36. R. W. Siegel, Hereditary endosymbiosis in *Paramecium bursaria*. *Exp. Cell Res.* **19**, 239–252 (1960).

37. S. Iwai, K. Fujiwara, T. Tamura, Maintenance of algal endosymbionts in *Paramecium bursaria*: A simple model based on population dynamics. *Environ. Microbiol.* **18**, 2435–2445 (2016).
38. Y. Kodama, M. Fujishima, Cycloheximide induces synchronous swelling of perialgal vacuoles enclosing symbiotic *Chlorella vulgaris* and digestion of the algae in the ciliate *Paramecium bursaria*. *Protist* **159**, 483–494 (2008).
39. C. F. Quispe, O. Sonderman, M. Khasin, W. R. Riekhof, J. L. Van Etten, K. W. Nickerson, Comparative genomics, transcriptomics, and physiology distinguish symbiotic from free-living *Chlorella* strains. *Algal Res.* **18**, 332–340 (2016).
40. B. A. Levenko, Y. K. Chemeris, P. S. Venediktov, Changes in the content of chlorophyll *a* spectral forms in synchronous culture and during nitrogen starvation of *Chlorella*. *Biochem. Physiol. Pflanz.* **180**, 157–162 (1985).
41. J. L. Gómez Pinchetti, E. del Campo Fernández, P. Moreno Díez, G. G. Reina, Nitrogen availability influences the biochemical composition and photosynthesis of tank-cultivated *Ulva rigida* (Chlorophyta). *J. Appl. Phycol.* **10**, 383–389 (1998).
42. C. García-Ferris, A. de los Ríos, C. Ascaso, J. Moreno, Correlated biochemical and ultrastructural changes in NITROGEN-STARVEDEUGLENA *GRACILIS*[1]. *J. Phycol.* **32**, 953–963 (1996).
43. S. Schmollinger, T. Mühlhaus, N. R. Boyle, I. K. Blaby, D. Casero, T. Mettler, J. L. Moseley, J. Kropat, F. Sommer, D. Strenkert, D. Hemme, M. Pellegrini, A. R. Grossman, M. Stitt, M. Schroda, S. S. Merchant, Nitrogen-sparing mechanisms in *Chlamydomonas* affect the transcriptome, the proteome, and photosynthetic metabolism. *Plant Cell* **26**, 1410–1435 (2014).
44. D. H. Turpin, Effects of inorganic N availability on algal photosynthesis and carbon metabolism. *J. Phycol.* **27**, 14–20 (1991).
45. L. Zhang, M. D. Cervantes, S. Pan, J. Lindsley, A. Dabney, G. M. Kapler, Transcriptome analysis of the binucleate ciliate *Tetrahymena thermophila* with asynchronous nuclear cell cycles. *Mol. Biol. Cell* **34**, rs1 (2023).
46. S. Y. Miyagishima, K. Suzuki, K. Okazaki, Y. Kabeya, Expression of the nucleus-encoded chloroplast division genes and proteins regulated by the algal cell cycle. *Mol. Biol. Evol.* **29**, 2957–2970 (2012).
47. K. Pecani, K. Lieberman, N. Tajima-Shirasaki, M. Onishi, F. R. Cross, Control of division in *Chlamydomonas* by cyclin B/CDKB1 and the anaphase-promoting complex. *PLOS Genet.* **18**, e1009997 (2022).
48. R. E. Hecky, P. Kilham, Nutrient limitation of phytoplankton in freshwater and marine environments: A review of recent evidence on the effects of enrichment. *Limnol. Oceanogr.* **33**, 796–822 (1988).
49. H. Schoffman, H. Lis, Y. Shaked, N. Keren, Iron–nutrient interactions within phytoplankton. *Front. Plant Sci.* **7**, 1223 (2016).
50. Y. Kodama, M. Fujishima, Symbiotic *Chlorella* sp. of the ciliate *Paramecium bursaria* do not prevent acidification and lysosomal fusion of host digestive vacuoles during infection. *Protoplasma* **225**, 191–203 (2005).
51. M. Tanaka, I. Miwa, Significance of photosynthetic products of symbiotic *Chlorella* to establish the endosymbiosis and to express the mating reactivity rhythm in *Paramecium bursaria*. *Zoolog. Sci.* **13**, 685–692 (1996).
52. Y. Kodama, S. Miyazaki, Autolysis of *Chlorella variabilis* in starving *Paramecium bursaria* help the host cell survive against starvation stress. *Curr. Microbiol.* **78**, 558–565 (2021).
53. E. Himi, T. Miyoshi-Akiyama, Y. Matsushima, I. Shiono, S. Aragane, Y. Hirano, G. Ikeda, Y. Kitaura, K. Kobayashi, D. Konno, A. Morohashi, Y. Noguchi, Y. Ominato, S. Shinbo, N. Suzuki, K. Takatsuka, H. Tashiro, Y. Yamada, K. Yamashita, N. Yoshino, M. Kitashima, S. Kotani, K. Inoue, A. Hino, H. Hosoya, Establishment of an unfed strain of *Paramecium bursaria* and analysis of associated bacterial communities controlling its proliferation. *Front. Microbiol.* **14**, 1036372 (2023).
54. W. J. Oswald, "Microalgae and wastewater treatment" in *Microalgal Biotechnology*, M. A. Borowitzka, L. J. Borowitzka, Eds. (Cambridge University Press, 1988), pp. 305–328.
55. B. S. Twining, S. B. Baines, The trace metal composition of marine phytoplankton. *Ann. Rev. Mar. Sci.* **5**, 191–215 (2013).
56. R. Geider, J. La Roche, Redfield revisited: Variability of C:N:P in marine microalgae and its biochemical basis. *Eur. J. Phycol.* **37**, 1–17 (2002).
57. J. Erez, A. Almogi-Labin, S. Avraham, On the life history of planktonic Foraminifera: Lunar reproduction cycle in *Globigerinoides* Sacculifer(Brady). *Paleoceanography* **6**, 295–306 (1991).
58. A. Tame, T. Maruyama, T. Ikuta, Y. Chikaraishi, N. O. Ogawa, M. Tsuchiya, K. Takishita, M. Tsuda, M. Hirai, Y. Takaki, N. Ohkouchi, K. Fujikura, T. Yoshida, mTORC1 regulates phagosome digestion of symbiotic bacteria for intracellular nutritional symbiosis in a deep-sea mussel. *Sci. Adv.* **9**, eadg8364 (2023).
59. E. Titlyanov, T. Titlyanova, V. Leletkin, J. Tsukahara, R. van Woesik, K. Yamazato, Degradation of zooxanthellae and regulation of their density in hermatypic corals. *Mar. Ecol. Prog. Ser.* **139**, 167–178 (1996).
60. D. A. Caron, A. W. H. Bé, O. R. Anderson, Effects of variations in light intensity on life processes of the planktonic foraminifer *Globigerinoides sacculiferin* laboratory culture. *J. Mar. Biol. Assoc. U.K.* **62**, 435–451 (1982).
61. A. W. H. Bé, O. R. Anderson, W. W. Faber, D. A. Caron, A. W. H. Be, Sequence of morphological and cytoplasmic changes during gametogenesis in the planktonic foraminifer *Globigerinoides sacculifer* (Brady). *Micropaleontology* **29**, 310 (1983).
62. M. E. Streams, C. R. Fisher, A. Fiala-Médioni, Methanotrophic symbiont location and fate of carbon incorporated from methane in a hydrocarbon seep mussel. *Mar. Biol.* **129**, 465–476 (1997).
63. A. Fiala-Médioni, Z. McKiness, P. Dando, J. Boulegue, A. Mariotti, A. Alayse-Danet, J. Robinson, C. Cavanaugh, Ultrastructural, biochemical, and immunological characterization of two populations of the mytilid mussel *Bathymodiolus azoricus* from the Mid-Atlantic Ridge: Evidence for a dual symbiosis. *Mar. Biol.* **141**, 1035–1043 (2002).
64. S. Kato, Laboratory culture and morphology of *Colacium vesiculosum* Ehrb. (Euglenophyceae). *Jpn. J. Phycol.* **30**, 63–67 (1982).
65. R. J. Porra, W. A. Thompson, P. E. Kriedemann, Determination of accurate extinction coefficients and simultaneous equations for assaying chlorophylls *a* and *b* extracted with four different solvents: Verification of the concentration of chlorophyll standards by atomic absorption *spectroscopy*. *Bioenergetics* **975**, 384–394 (1989).
66. J. Sambrook, E. F. Fritsch, T. Maniatis, *Molecular Cloning: A Laboratory Manual* (Cold Spring Harbor Laboratory Press, 2nd ed., 1989).
67. S. Chen, Y. Zhou, Y. Chen, J. Gu, fastp: An ultra-fast all-in-one FASTQ preprocessor. *Bioinformatics* **34**, i884–i890 (2018).
68. D. Kim, J. M. Paggi, C. Park, C. Bennett, S. L. Salzberg, Graph-based genome alignment and genotyping with HISAT2 and HISAT-genotype. *Nat. Biotechnol.* **37**, 907–915 (2019).
69. W. Li, A. Godzik, Cd-hit: A fast program for clustering and comparing large sets of protein or nucleotide sequences. *Bioinformatics* **22**, 1658–1659 (2006).
70. L. Fu, B. Niu, Z. Zhu, S. Wu, W. Li, CD-HIT: Accelerated for clustering the next-generation sequencing data. *Bioinformatics* **28**, 3150–3152 (2012).
71. M. G. Grabherr, B. J. Haas, M. Yassour, J. Z. Levin, D. A. Thompson, I. Amit, X. Adiconis, L. Fan, R. Raychowdhury, Q. Zeng, Z. Chen, E. Mauceli, N. Hacohen, A. Gnirke, N. Rhind, F. di Palma, B. W. Birren, C. Nusbaum, K. Lindblad-Toh, N. Friedman, A. Regev, Full-length transcriptome assembly from RNA-seq data without a reference genome. *Nat. Biotechnol.* **29**, 644–652 (2011).
72. S. R. Eddy, Accelerated profile HMM searches. *PLOS Comput. Biol.* **7**, e1002195 (2011).
73. B. Li, C. N. Dewey, RSEM: Accurate transcript quantification from RNA-Seq data with or without a reference genome. *BMC Bioinformatics* **12**, 323 (2011).
74. B. Buchfink, K. Reuter, H.-G. Drost, Sensitive protein alignments at tree-of-life scale using DIAMOND. *Nat. Methods* **18**, 366–368 (2021).
75. Y. Moriya, M. Itoh, S. Okuda, A. C. Yoshizawa, M. Kanehisa, KAAS: An automatic genome annotation and pathway reconstruction server. *Nucleic Acids Res.* **35**, W182–W185 (2007).
76. L. van der Maaten, G. Hinton, Visualizing data using t-SNE. *J. Mach. Learn. Res.* **9**, 2579–2605 (2008).
77. L. van der Maaten, Accelerating t-SNE using tree-based algorithms. *J. Mach. Learn. Res.* **15**, 3221–3245 (2014).
78. Y. Chen, L. Chen, A. T. L. Lun, P. L. Baldoni, G. K. Smyth, edgeR v4: Powerful differential analysis of sequencing data with expanded functionality and improved support for small counts and larger datasets. *Nucleic Acids Res.* **53**, gkaf018 (2025).

Acknowledgments: *P. bursaria* (NIES-2891) and *R. fusiformis* (NIES-123) were provided by the NIES through the NBRP of the MEXT, Japan. We also thank the members of the Miyagishima laboratory for insightful discussions. We would like to express gratitude to the reviewers who contributed to the improvement of this manuscript. **Funding:** This work was supported by the Japan Society for the Promotion of Science KAKENHI grant, Grant-in-Aid for JSPS Fellows 24KJ1163 (K.O.); Japan Society for the Promotion of Science KAKENHI grant, Grants-in-Aid for Scientific Research 24H00579 (S.-y.M.); and Japan Science and Technology Agency, JST-MIRAI Program JPMJMI22E1 (S.-y.M.). **Author contributions:** Conceptualization: Y.K., K.O., and S.-y.M. Methodology: K.O., S.H., and Y.K. Resources: Y.K. and K.O. Investigation: T.F., Y.K., K.O., and S.-y.M. Data curation: K.O., R.O., and S.-y.M. Validation: K.O., Y.K., and S.-y.M. Formal analysis: K.O., Y.K., R.O., and S.-y.M. Visualization: K.O. and S.-y.M. Writing—original draft: K.O. and S.-y.M. Writing—review and editing: K.O., T.F., S.H., Y.K., R.O., and S.-y.M. Project administration: K.O. and S.-y.M. Supervision: S.-y.M. Funding acquisition: K.O. and S.-y.M. **Competing interests:** The authors declare that they have no competing interests. **Data and materials availability:** All data needed to evaluate the conclusions in the paper are present in the paper and/or the Supplementary Materials. The raw RNA-seq data generated in this study are available under BioProject accession number PRJDB20461, https://ncbi.nlm.nih.gov/bioproject/?term=PRJDB20461.

Submitted 14 May 2025
Accepted 11 September 2025
Published 29 October 2025
10.1126/sciadv.adz0004
