# Supplementary material for: The closed nutrient recycling system in the Paramecium-Chlorella photosymbiosis contributes to survival under oligotrophic conditions
Source: Sci Adv. 2025 Oct 29;11(44):eadz0004. doi: 10.1126/sciadv.adz0004 (PMC12571070; doi:10.1126/sciadv.adz0004)
Supplement: Supplementary file 2 — Figs. S1 to S7 Legends for data S1 to S8 [file sciadv.adz0004_sm.pdf]

Science Advances
AAAS

Supplementary Materials for

The closed nutrient recycling system in the *Paramecium-Chlorella* photosymbiosis contributes to survival under oligotrophic conditions

Kaoru Okada *et al.*

Corresponding author: Kaoru Okada, kokada@nig.ac.jp; Shin-ya Miyagishima, smiyagis@nig.ac.jp

Sci. Adv. **11**, eadz0004 (2025)
DOI: 10.1126/sciadv.adz0004

The PDF file includes:

Figs. S1 to S7
Legends for data S1 to S8

Other Supplementary Material for this manuscript includes the following:

Data S1 to S8

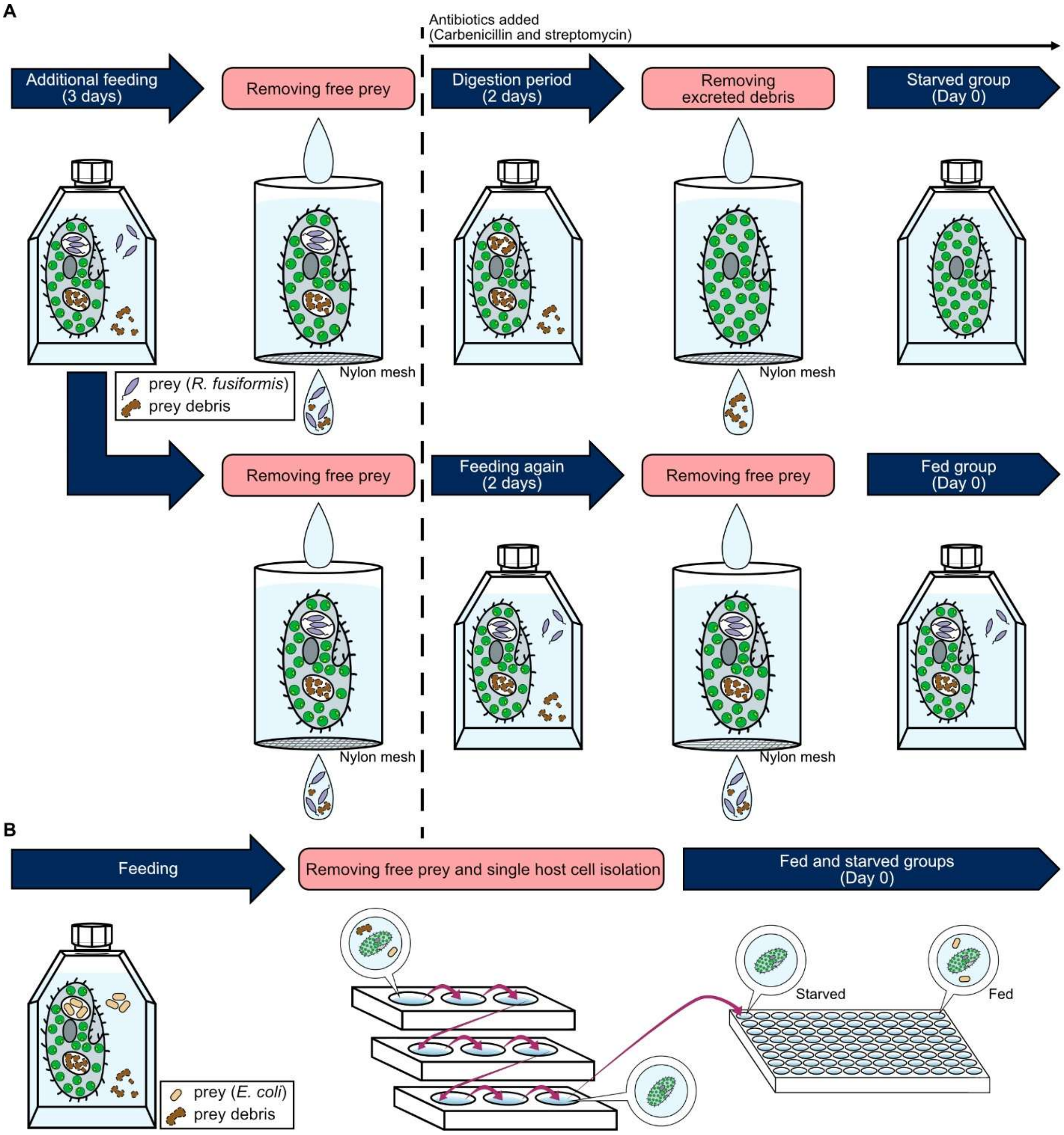

Fig. S1. Schematic illustration of the preparation of *P. bursaria* culture under starvation

(A) Standard protocol for comparison between the fed and starved cells. Before starvation, *P. bursaria* was cultivated for 3 days in the light with prey (*R. fusiformis*). To remove free prey from the medium, the culture was filtered through a 5-µm-pore nylon mesh. After that, *P. bursaria* cells were cultured for an additional 2 days in fresh medium without prey to ensure complete digestion of ingested prey. To eliminate undigestible debris excreted into the medium, the culture was filtered again, and *P. bursaria* cells were transferred to fresh medium without prey. This point was defined as day 0, from which the cells were cultured further. For comparison, the fed culture was prepared by adding *R. fusiformis* to the day 0 culture. **(B)** Protocol specific to Fig. 9, in which fed (with *E. coli* instead of *R. fusiformis*) and starved

cultures were started from a single *P. bursaria* cell. Single cells of *P. bursaria* cultured with *E. coli* prey were washed by pipetting on a blood cell reaction plate with fresh mAF-6ΔNPFe medium. After nine washes, the single cells were transferred individually to wells of a 96-well plate, and culture was initiated. This point was defined as day 0. For the fed group, *E. coli* was added as prey to the culture at day 0.

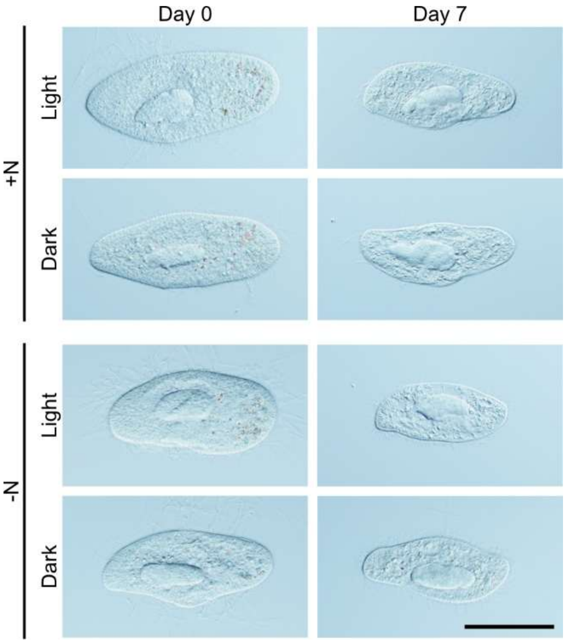

Fig. S2. Micrographs of aposymbiotic *P. bursaria* during starvation in nitrogen-replete or nitrogen-depleted inorganic media

Other results related to these cultures are shown in Fig. 1. Scale bar = 50 µm. As shown in Fig. 1, almost all cells had died by day 14; therefore, unlike the other cultures shown in Fig. 1, no images are available for days 14 and 21.

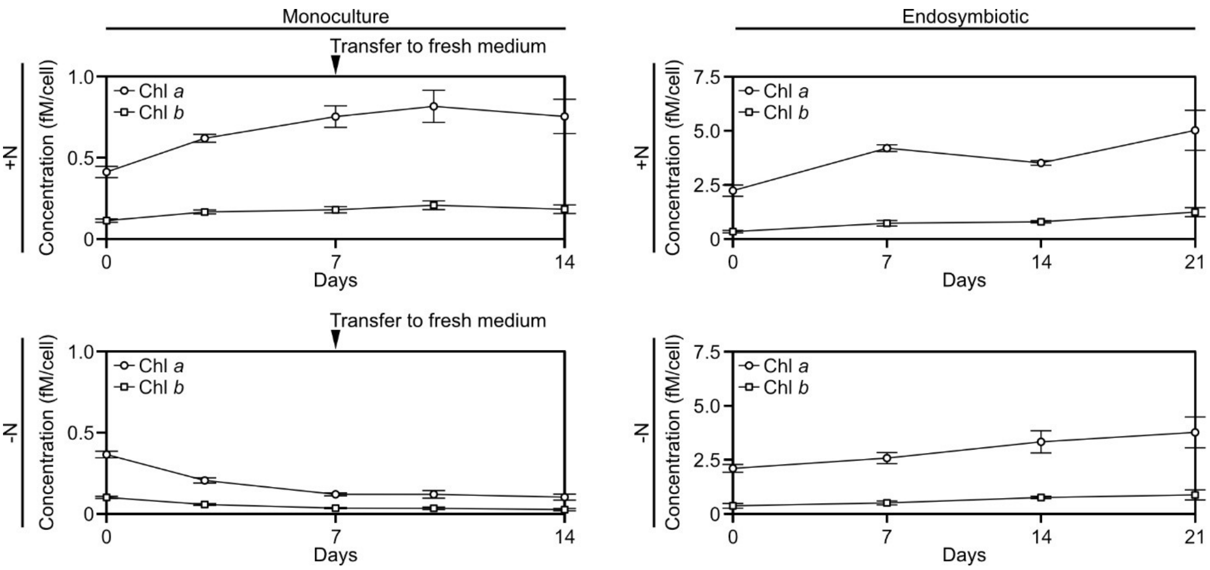

Fig. S3. Change in chlorophyll levels of *C. variabilis* in starved *P. bursaria* host cells and in monoculture

P. bursaria cells harbouring *C. variabilis* endosymbionts were subjected to starvation as shown in fig. S1 and Fig. 1, under light conditions, in nitrogen-replete (+N) and nitrogen-depleted (–N) media. Additionally, *C. variabilis* cells were monocultured under the same light conditions in nitrogen-replete (+N) and nitrogen-depleted (–N) media, as shown in Fig. 2. Changes in chlorophyll *a* and *b* contents per *C. variabilis* cell were examined. Means ± SD from four independent cultures are shown

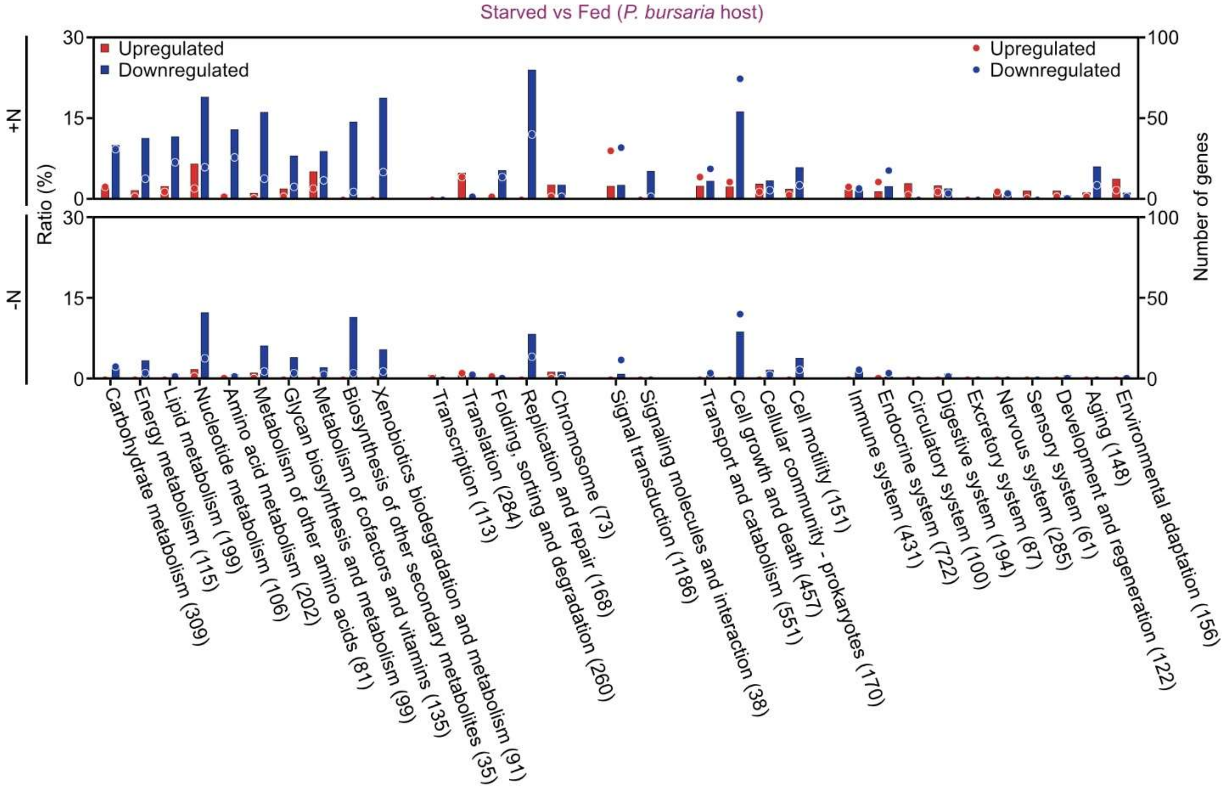
Starved vs Fed (P. bursaria host)
Upregulated
Downregulated
+N
-N
Ratio (%)
Number of genes
0
15
30
50
100
Carbohydrate metabolism (309)
Energy metabolism (115)
Lipid metabolism (199)
Nucleotide metabolism (106)
Amino acid metabolism (202)
Metabolism of other amino acids (81)
Glycan biosynthesis and metabolism (99)
Metabolism of cofactors and vitamins (135)
Biosynthesis of other secondary metabolites (35)
Xenobiotics biodegradation and metabolism (91)
Transcription (113)
Translation (284)
Folding, sorting and degradation (260)
Replication and repair (168)
Chromosome (73)
Signal transduction (1186)
Signaling molecules and interaction (38)
Transport and catabolism (551)
Cell growth and death (457)
Cellular community - prokaryotes (170)
Cell motility (151)
Immune system (431)
Endocrine system (722)
Circulatory system (100)
Digestive system (194)
Excretory system (87)
Nervous system (285)
Sensory system (61)
Development and regeneration (122)
Aging (148)
Environmental adaptation (156)

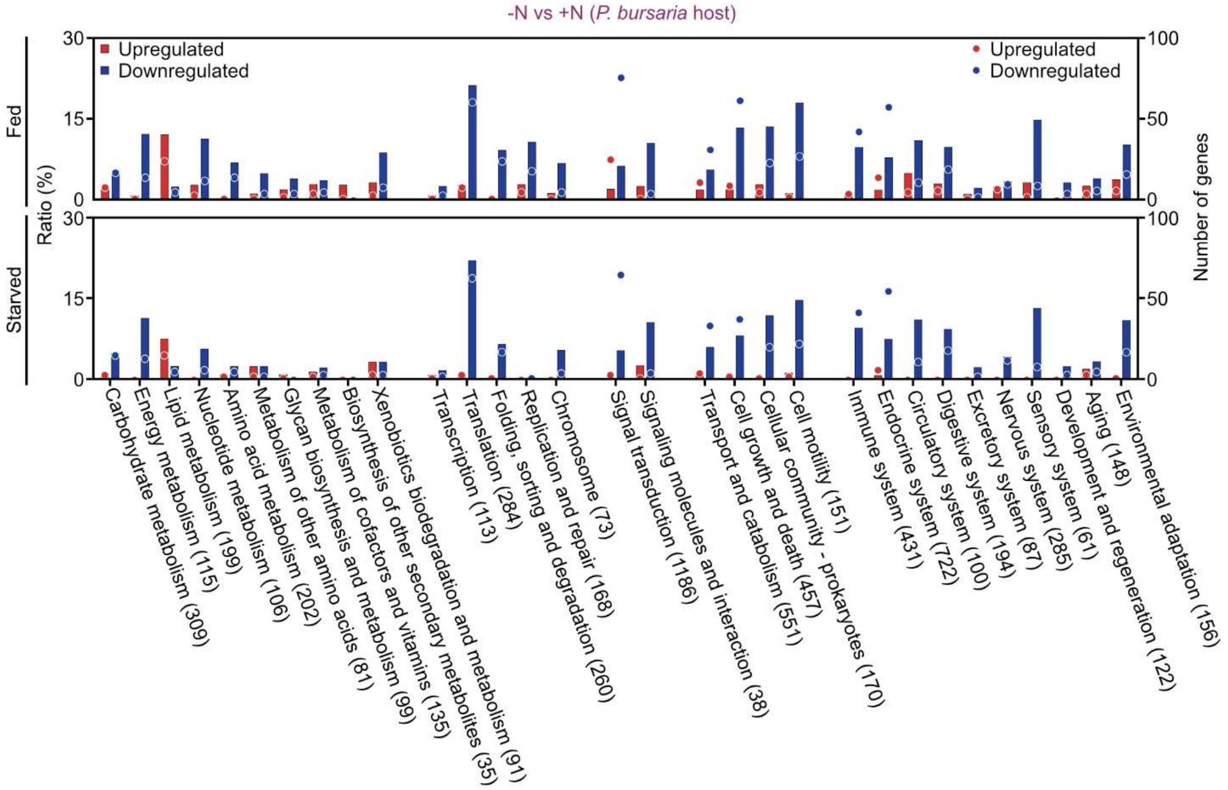
-N vs +N (P. bursaria host)
Upregulated
Downregulated
Fed
Starved
Ratio (%)
Number of genes
0
15
30
50
100
Carbohydrate metabolism (309)
Energy metabolism (115)
Lipid metabolism (199)
Nucleotide metabolism (106)
Amino acid metabolism (202)
Metabolism of other amino acids (81)
Glycan biosynthesis and metabolism (99)
Metabolism of cofactors and vitamins (135)
Biosynthesis of other secondary metabolites (35)
Xenobiotics biodegradation and metabolism (91)
Transcription (113)
Translation (284)
Folding, sorting and degradation (260)
Replication and repair (168)
Chromosome (73)
Signal transduction (1186)
Signaling molecules and interaction (38)
Transport and catabolism (551)
Cell growth and death (457)
Cellular community - prokaryotes (170)
Cell motility (151)
Immune system (431)
Endocrine system (722)
Circulatory system (100)
Digestive system (194)
Excretory system (87)
Nervous system (285)
Sensory system (61)
Development and regeneration (122)
Aging (148)
Environmental adaptation (156)

Fig. S4. KEGG functional classification of DEGs in *P. bursaria* cells harbouring *C. variabilis* endosymbionts under fed and starved conditions, and in nitrogen-replete and nitrogen-depleted media

P. bursaria cells harbouring *C. variabilis* endosymbionts were subjected to starvation as shown in fig. S1 and Fig. 1, under light conditions, in nitrogen-replete (+N) and nitrogen-depleted (–N) media. Differentially expressed genes (DEGs; FDR < 0.05 and $|\log_2$ fold change$| > 1$) were identified based on RNA-seq results from cells on day 3 of the starved or fed cultures in each medium, and were assigned to KEGG functional categories. Genes up- and down-regulated under starved (vs fed) or –N (vs +N) conditions are shown in red and blue, respectively. The circles indicate the number of up- or down-regulated genes, and the bars represent the percentage of these DEGs relative to the total number of genes assigned to each KEGG category. In the graphs, only categories that include more than 30 genes are shown. The IDs, read counts, and TPM values of the DEGs from the RNA-seq analysis are listed in datasets S1, S4, and S5.

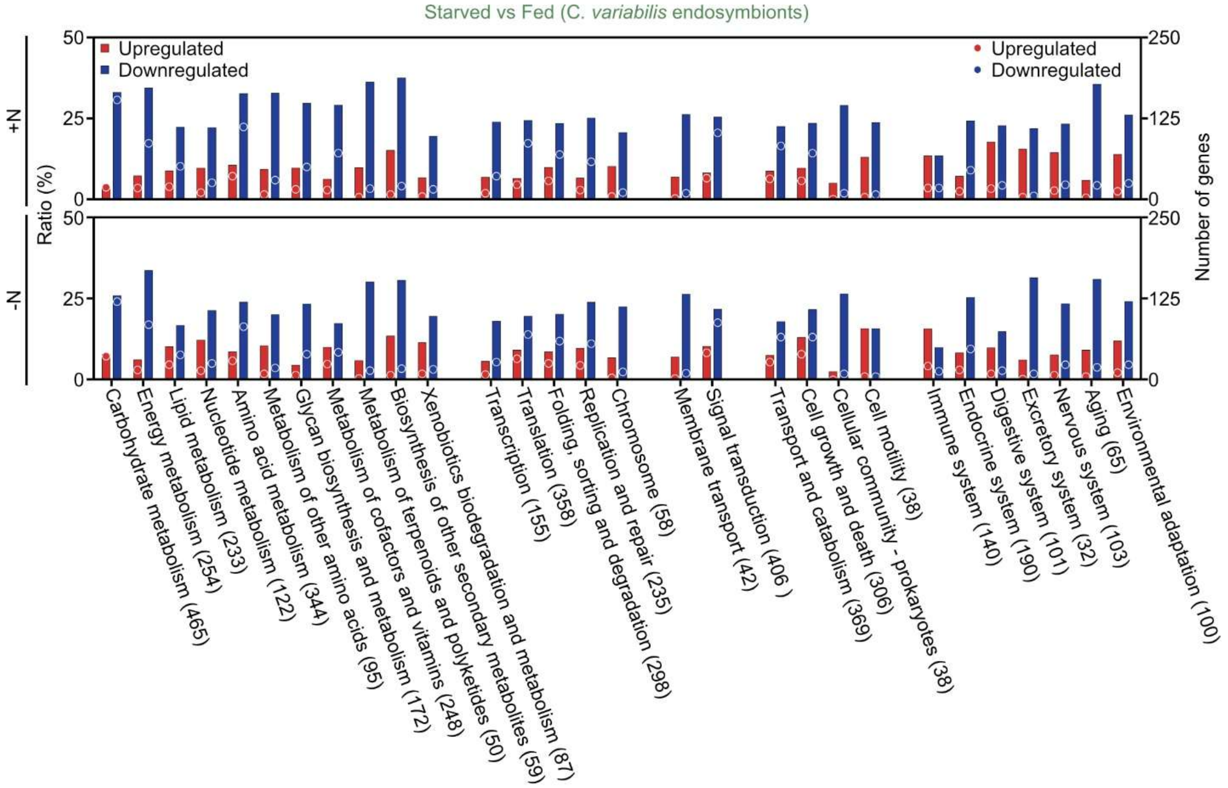
Starved vs Fed (C. *variabilis* endosymbionts)
Upregulated
Downregulated
Upregulated
Downregulated
+N
-N
Ratio (%)
Number of genes
50
25
0
250
125
0
Carbohydrate metabolism (465)
Energy metabolism (254)
Lipid metabolism (233)
Nucleotide metabolism (122)
Amino acid metabolism (344)
Metabolism of other amino acids (95)
Glycan biosynthesis and metabolism (172)
Metabolism of cofactors and vitamins (248)
Metabolism of terpenoids and polyketides (50)
Biosynthesis of other secondary metabolites (59)
Xenobiotics biodegradation and metabolism (87)
Transcription (155)
Translation (358)
Folding, sorting and degradation (298)
Replication and repair (235)
Chromosome (58)
Membrane transport (42)
Signal transduction (406)
Transport and catabolism (369)
Cell growth and death (306)
Cellular community - prokaryotes (38)
Cell motility (38)
Immune system (140)
Endocrine system (190)
Digestive system (101)
Excretory system (32)
Nervous system (103)
Aging (65)
Environmental adaptation (100)

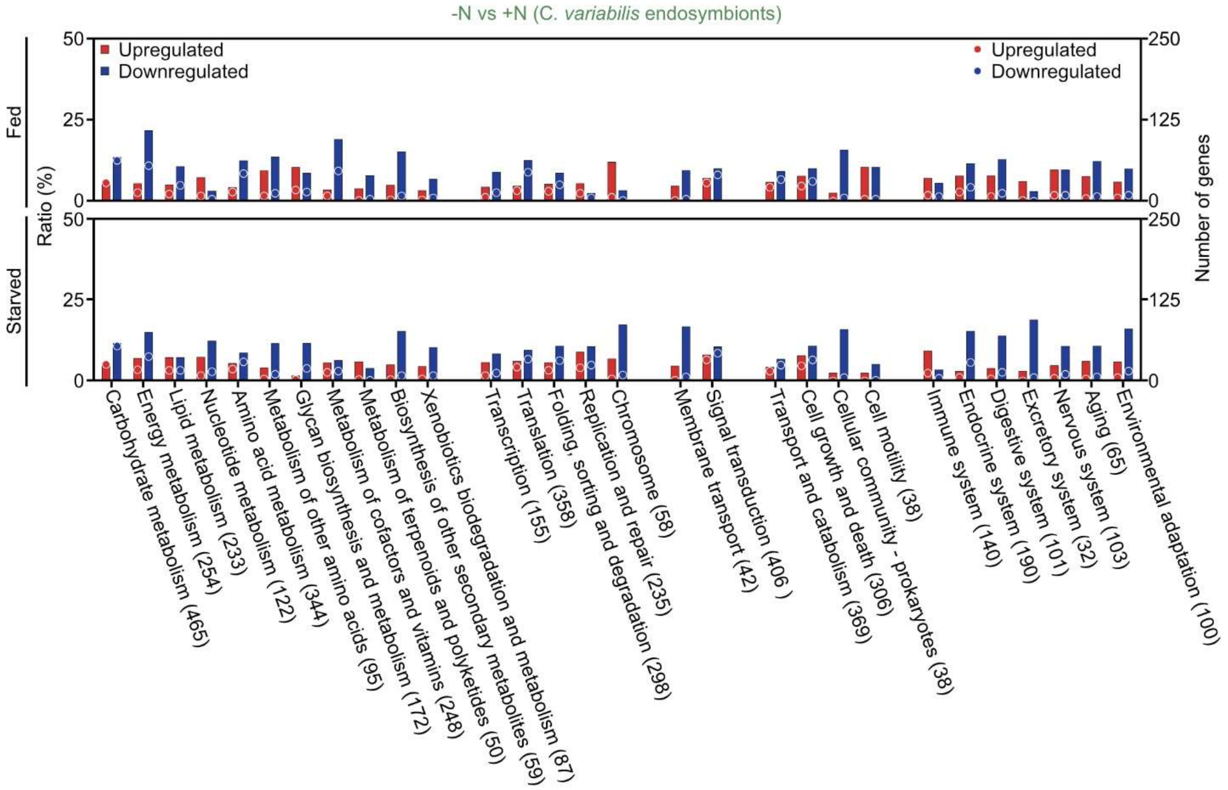
-N vs +N (C. *variabilis* endosymbionts)
Upregulated
Downregulated
Upregulated
Downregulated
Fed
Starved
Ratio (%)
Number of genes
50
25
0
250
125
0
Carbohydrate metabolism (465)
Energy metabolism (254)
Lipid metabolism (233)
Nucleotide metabolism (122)
Amino acid metabolism (344)
Metabolism of other amino acids (95)
Glycan biosynthesis and metabolism (172)
Metabolism of cofactors and vitamins (248)
Metabolism of terpenoids and polyketides (50)
Biosynthesis of other secondary metabolites (59)
Xenobiotics biodegradation and metabolism (87)
Transcription (155)
Translation (358)
Folding, sorting and degradation (298)
Replication and repair (235)
Chromosome (58)
Membrane transport (42)
Signal transduction (406)
Transport and catabolism (369)
Cell growth and death (306)
Cellular community - prokaryotes (38)
Cell motility (38)
Immune system (140)
Endocrine system (190)
Digestive system (101)
Excretory system (32)
Nervous system (103)
Aging (65)
Environmental adaptation (100)

Fig. S5. KEGG **functional classification of DEGs in *C. variabilis* endosymbionts in *P. bursaria* host cells under fed and starved conditions, and in nitrogen-replete and nitrogen-depleted media**

This figure is identical to Fig. S4, except that it shows DEGs from *C. variabilis* endosymbionts rather than from the *P. bursaria* host. The IDs, read counts, and TPM values of the DEGs from the RNA-seq analysis are listed in datasets S2, S6, and S7.

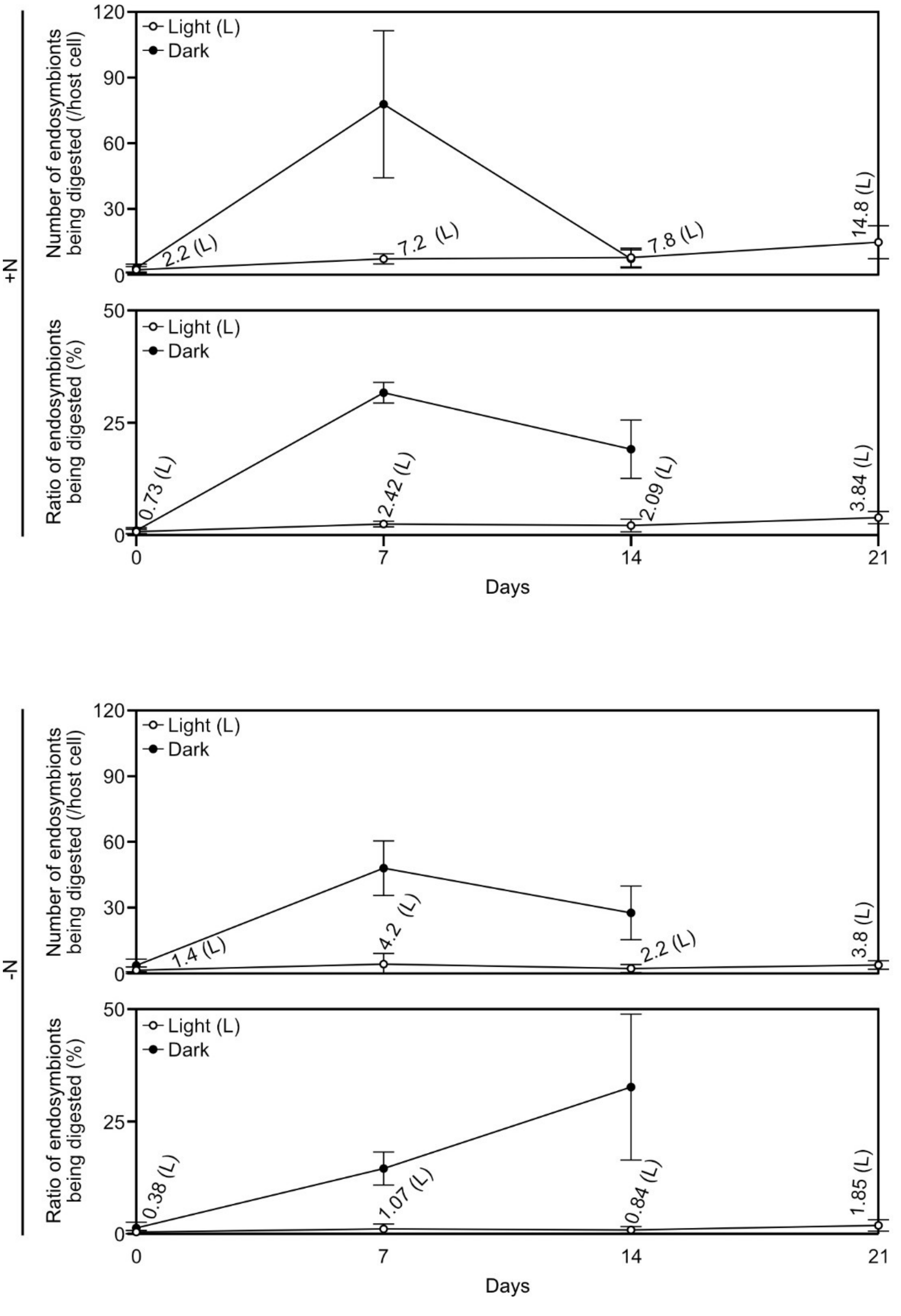

Fig. S6. Number and percentage of *C. variabilis* endosymbionts being digested in *P. bursaria* host cells during starvation

P. bursaria cells harbouring *C. variabilis* endosymbionts were subjected to starvation as shown in fig. S1 and Fig. 1. The number of *C. variabilis* endosymbionts being digested per host cell and their percentage within the *C. variabilis* population in host cells under each culture condition were determined by microscopy. As shown in Fig. 1, *P. bursaria* cells in the dark died by day

21; thus, no data are available for the dark condition on day 21. For the light conditions, the values are also indicated in the graphs.

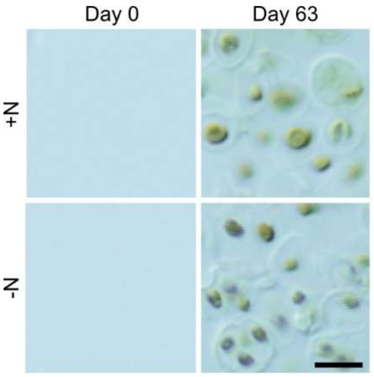

Fig. S7. Micrographs of debris from digested *C. variabilis* endosymbionts excreted by *P. bursaria* hosts during starvation culture

P. bursaria cells harbouring *C. variabilis* endosymbionts were subjected to starvation in nitrogen-replete (+N) and nitrogen-depleted (–N) inorganic media, as shown in Fig. 7. In the later stage of the culture, sediment was observed at the bottom of the culture flasks. Sediment samples were collected from the bottom of the flasks on day 63. For comparison, medium from the bottom of the flasks on day 0 was also collected and examined by microscopy. Scale bar = 5 µm.

Data S1. (separate file)
Read counts and TPM values of genes in the *P. bursaria* host under each culture condition.

Data S2. (separate file)
Read counts and TPM values of genes in the *C. variabilis* endosymbiont under each culture condition.

Data S3. (separate file)
List of gene IDs shown in Figs. 4 and 5.

Data S4. (separate file)
List of differentially expressed genes in the *P. bursaria* host.

Data S5. (separate file)
KEGG classification of differentially expressed genes in *P. bursaria* host.

Data S6. (separate file)
List of differentially expressed genes in the *C. variabilis* endosymbiont.

Data S7. (separate file)
KEGG classification of differentially expressed genes in *C. variabilis* endosymbiont.

Data S8. (separate file)
Sequences of primers used in this study.
